# A Peptide‐Induced Self‐Cleavage Reaction Initiates the Activation of Tyrosinase

**DOI:** 10.1002/anie.201901332

**Published:** 2019-04-17

**Authors:** Ioannis Kampatsikas, Aleksandar Bijelic, Matthias Pretzler, Annette Rompel

**Affiliations:** ^1^ Universität Wien Fakultät für Chemie Institut für Biophysikalische Chemie Althanstraße 14 1090 Wien Austria

**Keywords:** activating peptides, crystal structures, maturation agents, polyphenol oxidases, self-cleaving peptides

## Abstract

The conversion of inactive pro‐polyphenol oxidases (pro‐PPOs) into the active enzyme results from the proteolytic cleavage of its C‐terminal domain. Herein, a peptide‐mediated cleavage process that activates pro‐*Md*PPO1 (*Malus domestica*) is reported. Mass spectrometry, mutagenesis studies, and X‐ray crystal‐structure analysis of pro‐*Md*PPO1 (1.35 Å) and two separated C‐terminal domains, one obtained upon self‐cleavage of pro‐*Md*PPO1 and the other one produced independently, were applied to study the observed self‐cleavage. The sequence Lys 355–Val 370 located in the linker between the active and the C‐terminal domain is indispensable for the self‐cleavage. Partial introduction (Lys 352–Ala 360) of this peptide into the sequence of two other PPOs, *Md*PPO2 and aurone synthase (*Cg*AUS1), triggered self‐cleavage in the resulting mutants. This is the first experimental proof of a self‐cleavage‐inducing peptide in PPOs, unveiling a new mode of activation for this enzyme class that is independent of any external protease.

Tyrosinases (TYRs, EC 1.14.18.1 and EC 1.10.3.1) and catechol oxidases (COs, EC 1.10.3.1) are type III copper‐containing metalloenzymes that constitute the class of polyphenol oxidases (PPOs).^1^, ^2^ PPOs are present in archaea, bacteria, fungi, plants, and animals.^3^, ^4^, ^5^, ^6^ To date, plant, fungal, and bacterial PPOs have been reported to exist in both their pro‐ (or latent) and active form in vivo.^7^, ^8^, ^9^, ^10^ More specifically, plant PPOs are expressed as pro‐enzymes (55–65 kDa) consisting of an enzymatically active (40–45 kDa) and a C‐terminal domain (15–19 kDa).^11^, ^12^ The C‐terminal domain plays a significant role in the regulation of the enzyme activity inside the cell by shielding the catalytic pocket of the active centre, and in addition it provides an indispensable motif for the accurate folding of the active domain.^13^ PPOs are in general activated by the removal of their C‐terminal domain but the in vivo activation mechanism of PPOs is still widely unknown, with the exception of three insect PPOs, which are activated by a complex serine proteinase cascade.^14^ It is widely accepted that pro‐PPOs are activated by a proteolytic reaction followed by the spatial detachment of the C‐terminal domain from the active protein.^12^, ^15^ PPOs such as apple and mushroom tyrosinases have been reported to be activated in vitro by common proteases;^16^, ^17^ however, in both cases, the C‐terminal domain was not specifically cleaved but rather completely digested by the respective proteases (trypsin and proteinase K). Owing to the lack of knowledge about the activation process in vivo, the detergent sodium dodecyl sulfate (SDS) is currently used predominantly to activate pro‐PPOs in vitro.^16^, ^17^, ^18^ The detergent is believed to induce structural changes within the enzyme that make its active centre more accessible for incoming substrates. Herein, we investigated the activation of PPO1 from *Malus domestica* (*Md*PPO1) and present a novel activation mode for plant PPOs driven by self‐cleavage, which is independent of external proteases or any other harsh conditions (e.g., SDS). Extensive SDS‐PAGE‐based investigations, mutagenesis experiments, mass spectrometry, and X‐ray crystal‐structure analysis were applied to explore the activation process of *Md*PPO1, leading to the identification of a peptide that is located in the linker region between the active and the C‐terminal domain and is indispensable for the self‐cleavage of the enzyme.

The pro‐form of *Md*PPO1 was recombinantly overexpressed in *E. coli*, purified, and finally subjected to crystallization.^16^, ^19^ During initial crystallization attempts with pro‐*Md*PPO1, only high‐quality crystals of the C‐terminal domain (C_cleaved_‐domain) were obtained (1.35 Å resolution, PDB No. 6ELT; Figure 1 B). Crystallization of the pro‐enzyme was only possible by fast processing (i.e., crystallization immediately after the last purification step) to avoid cleavage of the full‐length protein as much as possible, which finally led to the X‐ray structure of pro‐*Md*PPO1 (1.35 Å resolution, PDB No. 6ELS; Figure 1 A). Therefore, it was suspected that the pro‐enzyme undergoes self‐cleavage, severing the C‐terminal domain from the pro‐enzyme. We recently observed a similar process in apricot PPO, where the enzyme was spontaneously activated upon prolonged storage.^20^ The assumed self‐cleavage of *Md*PPO1 was confirmed by SDS‐PAGE of pro‐enzyme solutions incubated at 4 °C for 20 days, indicating the activation of the pro‐enzyme into its separated active and C‐terminal domains (Figure S1; see the Supporting Information for experimental details). To exclude the possibility that the observed self‐cleavage was caused by contaminations originating from the expression host *E. coli*, fresh *Md*PPO1 enzyme was incubated with lysate of *E. coli*, which did not infer any change to the cleavage of *Md*PPO1 (Figure S2). Interestingly, the enzyme retained its latency even after complete cleavage as it did not show any activity on mono‐ or diphenolic substrates. Thus it seems that the C‐terminal domain stays attached to the main domain, presumably because of strong electrostatic interactions between the two domains as indicated by PISA^21^ analysis (33 hydrogen bonds and 13 salt bridges). It was concluded that the observed cleavage converts the pro‐enzyme into a pre‐active stage that still requires the spatial removal of the C‐terminal domain (e.g., by SDS or high salt concentrations, see the Supporting Information and Figure S3) in order to achieve full enzymatic activity.^16^

**Figure 1:**
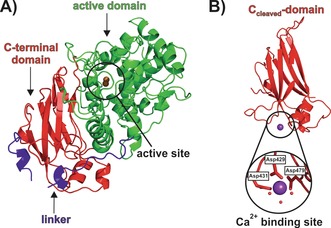
Crystal structure of pro‐*Md*PPO1 (PDB No. 6ELS) and the C_cleaved_‐domain (PDB No. 6ELT). A) The overall structure of pro‐*Md*PPO1. The main domain is shown in green, the C‐terminal domain in red, and the linker that connects the main and C‐terminal domains in blue. Owing to the absence of electron density, a part (Ala 349–Val 359) of the loop region within the C‐terminal domain is missing. B) The overall structure of the C_cleaved_‐domain with the Ca^2+^ binding site. Ca^2+^ (purple sphere) is coordinated by three aspartate residues (shown in stick mode) and three water molecules depicted as small red spheres.

The cleavage site was determined by high‐resolution electrospray ionization mass spectrometry (ESI‐MS), which showed that the protein is not just cleaved at one single peptide bond but rather within a sequence of four contiguous peptide bonds (Ser 366–Ser 367–Ser 368–Lys 369–Val 370; Figures 2 and S4). A similar cleavage behaviour was observed for walnut tyrosinase purified from the natural source, which is also activated by peptide cleavage within four amino acids (Pro 342–Thr 343–Pro 344–Arg 345–Lys 346),^22^ indicating that this is a general activation reaction of plant PPOs. The crystal structure of the C_cleaved_‐domain starts at residue Lys 369 and ends at Ser 504 (Figure S5), indicating that the C‐terminal domain remains stable upon self‐cleavage. To corroborate this result, an orthogonal experiment was performed, where the sole C‐terminal domain was recombinantly overexpressed (C_sole_‐domain) and crystallized. The resulting C_sole_‐domain structure (1.05 Å resolution, PDB No. 6ELV) confirmed the autonomous stability and independent folding of the C‐terminal domain as its structure did not differ from that of the C_cleaved_‐domain (C_α_ RMSD of 0.494 Å, 562 matched atoms). Structural analysis of the separated C‐terminal domains, C_cleaved_ and C_sole_, revealed a metal‐binding site. The bound metal was identified as Ca^2+^ based on the composition of the used expression media and buffers, the interacting amino acids, the binding geometry, and the presence of anomalous signal. This binding site is absent in the C‐terminal domain still attached to the pro‐enzyme (Figure 1). The core structure, and especially the active‐site region of pro‐*Md*PPO1, resembles those of other structurally known plant PPOs very closely, for example, tyrosinase from *Juglans regia* (*Jr*TYR, PDB No. 5CE9, sequence identity 66.6 %),^23^, ^24^ catechol oxidase from *Ipomoea batatas* (*Ib*CO, PDB No. 1BT3, sequence identity 53.0 %),^25^ and aurone synthase from *Coreopsis grandiflora* (*Cg*AUS1, PDB No. 4Z11, sequence identity 43.0 %).^11^, ^26^, ^27^

**Figure 2:**
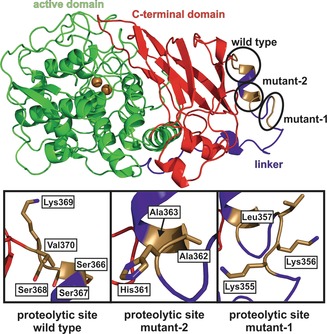
The different cleavage sites of wild‐type *Md*PPO1, mutant‐1, and mutant‐2. The wild type is cleaved within the sequence Ser 366–Ser 367–Ser 368–Lys 369–Val 370, mutant‐1 within Lys 355–Lys 356–Lys 357, and mutant‐2 within His 361–Ala 362–Ala 363.

To understand the self‐cleavage process in greater detail, a series of spectroscopic and biochemical experiments were performed to complement the X‐ray crystallographic study. Self‐cleavage was examined at different temperatures and at different pH values, revealing that the reaction was fastest at 37 °C and a pH value of 7 (Figures S6 and S7). Moreover, different protease inhibitors were applied in an attempt to inhibit the cleavage process. Two serine protease inhibitors (phenylmethylsulfonyl fluoride and benzamidine hydrochloride), an aspartyl protease inhibitor (pepstatin A), the metalloprotease inhibitor ethylenediaminetetraacetic acid (EDTA), and a commercially available mixture of several protease inhibitors (SigmaFAST) were tested (Figures S8 and S9). However, none of these inhibitors were able to inhibit the activation completely (see the Supporting Information). Subsequently, mutagenesis was applied to inhibit the self‐cleavage reaction of *Md*PPO1. The cleavage sequence Ser 366–Ser 367–Ser 368–Lys 369–Val 370 (Figure 2) was mutated to Ile 367–Asp 368–Gly 369–Arg 370 (*Md*PPO1‐mutant‐1), but the self‐cleavage was surprisingly not prevented. ESI‐MS analysis of *Md*PPO1‐mutant‐1 (upon cleavage) indicated a relocation of the cleavage site to the sequence Lys 355–Lys 356–Leu 357, representing a cleavage site shift by eleven amino acids towards the N‐terminus (Figures 2 and S10). Thus a second mutant (*Md*PPO1‐mutant‐2) was prepared by mutating both of the above identified cleavage sites (Ser 366–Ser 367–Ser 368–Lys 369–Val 370 and Lys 355–Lys 356–Leu 357 to Ile 367–Asp 368–Gly 369–Arg 370 and Gly 355–Ala 356–Gly 357, respectively; see Table S1). The cleavage reaction was again not stopped, and ESI‐MS revealed a third cleavage site comprising the peptide bonds His 361–Ala 362–Ala 363, which is located between the cleavage sites of the wild type and *Md*PPO1‐mutant‐1 (Figures 2 and S11). These observations indicate that the mode of action cannot be explained by a common sequence‐specific proteolytic reaction.

To gain further insight into the self‐cleavage reaction, a homology model of the isoenzyme *Md*PPO2^16^ was prepared and compared with the crystal structure of *Md*PPO1. *Md*PPO2 was chosen for comparison as it originates from the same organism but does not exhibit self‐cleavage (Figures S12 and S13). The structural comparison revealed one significant difference within the linker that connects the active and the C‐terminal domain (Figure 3 A). *Md*PPO1 contains a long and very exposed peptide (Lys 352–Val 370), which harbours all three detected cleavage sites, whereas the corresponding region in *Md*PPO2 is significantly smaller (Lys 350–Leu 360; Figure 3 A, D). A large part of this peptide is not obvious in the crystal structure of *Md*PPO1 owing to a lack of electron density in this region. However, it is still attached to *Md*PPO1 as confirmed by ESI‐MS.^16^ Therefore, the missing part was modelled with the software MODELLER.^28^ To confirm the involvement of the identified peptide in the self‐cleavage process, a mutant of *Md*PPO1 was prepared by deleting most of the peptide sequence (Lys 355–Val 370; Figure 3 B). The resulting mutant *Md*PPO1(−) was soluble and still enzymatically active as it accepted mono‐ and diphenolic substrates; however, it remained intact and did not exhibit self‐cleavage even after 14 weeks (Figures S12 and S13). This result confirmed that the identified peptide is indispensable for the self‐cleavage reaction of *Md*PPO1.

**Figure 3:**
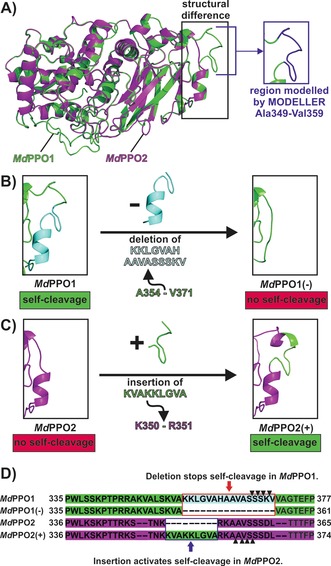
Structural comparison of *Md*PPO1, *Md*PPO2, and their respective mutants. A) Superposition of the crystal structure of *Md*PPO1 (green cartoon) and the homology model of *Md*PPO2 (magenta cartoon), which was prepared by using the SWISS‐MODEL Server.^29^ The black rectangle highlights a region of the linker where the isoenzymes differ significantly. The inset on the right indicates the region Ala 349–Val 359 of *Md*PPO1, which is missing in the structure owing to a lack of electron density and was therefore modelled with the software MODELLER^28^ (blue cartoon). B) The effect of the deletion of the peptide Lys 355–Val 370 (cyan cartoon) from the sequence of *Md*PPO1 (green cartoon) on the region highlighted in (A). The resulting mutant *Md*PPO1(−) does not exhibit self‐cleavage. C) The effect of the insertion of the peptide Lys 352–Ala 360 (KVAKKLGVA) from *Md*PPO1 (green cartoon) into the sequence of *Md*PPO2 (magenta cartoon) on the region highlighted in (A). The insertion converts the stable *Md*PPO2 into a self‐cleaving enzyme, mutant *Md*PPO2(+). D) Primary structures of *Md*PPO1, *Md*PPO2, and the respective mutants. The Figure highlights which part of the sequence was deleted from *Md*PPO1 to produce *Md*PPO1(−) and which sequence part was added to *Md*PPO2 to obtain *Md*PPO2(+). The black triangles indicate the respective cleavage sites in *Md*PPO1 and *Md*PPO2(+). E: glutamic acid, H: histidine, N: asparagine, D: aspartic acid, T: threonine, G: glycine, F: phenylalanine, V: valine, R: arginine, L: leucine, K: lysine, P: proline, A: alanine, S: serine, W: tryptophan.

To further confirm the self‐cleavage‐inducing role of this peptide, we attempted to induce self‐cleavage in *Md*PPO2 by inserting a part of the peptide (Lys 352–Val 370) into its sequence. The decision to introduce only a part of the sequence (9 amino acids) instead of the whole peptide (18 amino acids) was made for structural reasons as it was aimed to (structurally) adapt the length of the peptide to that found in *Md*PPO1 (Figure 3 D). For this reason, the sequence Lys 352–Ala 360 from *Md*PPO1 was introduced in between Lys 350 and Arg 351 of *Md*PPO2 (Figures 3 C, D and S12). The resulting mutant *Md*PPO2(+) indeed underwent self‐cleavage as evidenced by SDS‐PAGE (Figure S13). The cleavage site of *Md*PPO2(+) was determined to be Ala 362–Ala 363–Val 364–Ser 365–Ser 366 by ESI‐MS (Figure S14). Interestingly, although the insert (Lys 352–Ala 360) contains a cleavage site of *Md*PPO1 (Lys 355–Lys 356–Leu 357; Figure 2), *Md*PPO2(+) was cleaved at a region belonging to the original sequence of *Md*PPO2, which is located four amino acids downstream of the introduced peptide (Figure 3 D). This result provides further indication that the here described self‐cleavage does not depend on a specific sequence recognition. To confirm the generality of the self‐cleavage‐inducing role of this peptide for plant PPOs, another plant PPO incapable of self‐activation, aurone synthase (*Cg*AUS1), was mutated similarly to *Md*PPO2 by inserting the peptide sequence Lys 352–Ala 360 of *Md*PPO1 in between Ala 369 and Thr 370 of *Cg*AUS1 (Figure S12). The resulting *Cg*AUS1 mutant *Cg*AUS1(+) also showed self‐cleavage activity, and ESI‐MS revealed three proteolytic sites, namely Lys 374–Leu 375, Gly 376–Val 377, and Ala 378–Thr 379 (Figure S15). It therefore appears that the crucial peptide of *Md*PPO1 is able to induce self‐cleavage in different plant PPOs and not only in isoenzymes (*Md*PPO1 and *Md*PPO2) originating from the same organism. Moreover, pro‐*Md*PPO1 was incubated with different amounts of C_sole_‐domain to investigate whether the C‐terminal domain plays a role in the self‐cleavage process. The results clearly demonstrate that the addition of (external) C_sole_‐terminal domain significantly increases the self‐cleavage rate as with increasing concentration of external C_sole_‐domain, the pro‐enzyme is cleaved into its active and C‐terminal domain faster (Figure S16). Further mutagenesis experiments are summarized in the Supporting Information (Table S1).

In summary, we have recombinantly produced and successfully crystallized the pro‐form of *Md*PPO1 and two versions of its C‐terminal domain, one obtained after self‐cleavage (C_cleaved_) and the other one as an independently expressed domain (C_sole_). Protease inhibitors and mutations of the cleavage sites did not prevent the self‐cleavage, indicating the high tendency of *Md*PPO1 to undergo self‐cleavage. However, the deletion of the peptide Lys 355–Val 370 deactivated the self‐cleavage reaction in *Md*PPO1. On the other hand, partial insertion of this peptide (Lys 352–Ala 360) into *Md*PPO2 and *Cg*AUS1 converted the two stable enzymes into self‐cleaving PPOs. These findings represent the first evidence that PPOs undergo self‐cleavage for activation and reveal a novel mechanism that is independent of external proteases. This represents an important contribution to the field of protein (pro‐enzyme) activation as it contradicts the general assumption that PPOs are activated by external proteases, and could therefore explain the futility of the search for external proteases as the activating agents for most PPOs.

## Experimental Section

Detailed descriptions of the experiments are provided in the Supporting Information. For the crystallization of pro‐*Md*PPO1 and the C_sole_‐domain, enzymes were heterologously expressed and purified by affinity chromatography as described previously for pro‐*Md*PPO1.^16^ For the design and production of the mutants, the plasmids coding for pro‐*Md*PPO1, pro‐*Md*PPO2, and pro‐*Cg*AUS1 were used as templates. Pro‐*Md*PPO1 as well as the C_cleaved_‐ and C_sole_‐domains were crystallized, and the structures were determined by the molecular replacement method. Extensive SDS‐PAGE experiments were performed with different protease inhibitors at different temperatures and over a wide pH range. The exact self‐cleavage sites were determined by high‐resolution ESI‐MS.

## Conflict of interest

The authors declare no conflict of interest.

## Supporting information

As a service to our authors and readers, this journal provides supporting information supplied by the authors. Such materials are peer reviewed and may be re‐organized for online delivery, but are not copy‐edited or typeset. Technical support issues arising from supporting information (other than missing files) should be addressed to the authors.

Supplementary

## References

[1] E. I. Solomon , D. E. Heppner , E. M. Johnston , J. W. Ginsbach , J. Cirera , M. Qayyum , M. T. Kieber-Emmons , C. H. Kjaergaard , R. G. Hadt , L. Tian , Chem. Rev. 2014, 114, 3659–3853. 24588098 10.1021/cr400327t PMC4040215

[2] C. Kaintz , S. G. Mauracher , A. Rompel in Adv. Protein Chem. Struct. Biol. (Ed.: C. Z. Christov), Elsevier, Amsterdam, 2014, pp. 1–35, 10.1016/bs.apcsb.2014.07.001. 25458353

[3] L. T. Tran , J. S. Taylor , C. P. Constabel , BMC Genomics 2012, 13, 395. 22897796 10.1186/1471-2164-13-395 PMC3472199

[4] A. M. Mayer , Phytochemistry 2006, 67, 2318–2331. 16973188 10.1016/j.phytochem.2006.08.006

[5] M. Pretzler , A. Bijelic , A. Rompel in Ref. Module Chem. Mol. Sci. Chem. Eng., Elsevier, Amsterdam, 2015, 10.1016/B978-0-12-409547-2.11521-5.

[6] M. Pretzler , A. Rompel , Inorg. Chim. Acta 2018, 481, 25–31.

[7] R. Yoruk , M. R. Marshall , J. Food Biochem. 2003, 27, 361–422.

[8] M. Fairhead , L. Thöny-Meyer , FEBS J. 2010, 277, 2083–2095. 20345903 10.1111/j.1742-4658.2010.07621.x

[9] S. G. Mauracher , C. Molitor , C. Michael , M. Kragl , A. Rizzi , A. Rompel , Phytochemistry 2014, 99, 14–25. 24461779 10.1016/j.phytochem.2013.12.016 PMC3969299

[10] C. Molitor , S. G. Mauracher , S. Pargan , R. L. Mayer , H. Halbwirth , A. Rompel , Planta 2015, 242, 519–537. 25697287 10.1007/s00425-015-2261-0 PMC4540782

[11] C. Molitor , S. G. Mauracher , A. Rompel , Proc. Natl. Acad. Sci. USA 2016, 113, E1806–E1815. 10.1073/pnas.1523575113 PMC4822611 26976571

[12] W. H. Flurkey , J. K. Inlow , J. Inorg. Biochem. 2008, 102, 2160–2170. 18829115 10.1016/j.jinorgbio.2008.08.007

[13] L. L. Moe , S. Maekawa , Y. Kawamura-Konishi , Appl. Microbiol. Biotechnol. 2015, 99, 5499–5510. 25904132 10.1007/s00253-015-6597-y

[14] A. Lu , Q. Zhang , J. Zhang , B. Yang , K. Wu , W. Xie , Y.-X. Luan , E. Ling , Front. Physiol. 2014, 5, 252. 25071597 10.3389/fphys.2014.00252 PMC4092376

[15] G. Faccio , M. Arvas , L. Thöny-Meyer , M. Saloheimo , J. Inorg. Biochem. 2013, 121, 37–45. 23333757 10.1016/j.jinorgbio.2012.12.006

[16] I. Kampatsikas , A. Bijelic , M. Pretzler , A. Rompel , Sci. Rep. 2017, 7, 8860. 28821733 10.1038/s41598-017-08097-5 PMC5562730

[17] M. Pretzler , A. Bijelic , A. Rompel , Sci. Rep. 2017, 7, 1810. 28500345 10.1038/s41598-017-01813-1 PMC5431950

[18] H. J. Martin , I. Kampatsikas , R. Oost , M. Pretzler , E. Al-Sayed , A. Roller , G. Giester , A. Rompel , N. Maulide , Chem. Eur. J. 2018, 24, 15756–15760. 30113748 10.1002/chem.201803785 PMC6220842

[19] I. Kampatsikas , A. Bijelic , M. Pretzler , A. Rompel , Acta Crystallogr. Sect. F 2017, 73, 491–499. 10.1107/S2053230X17010822 PMC5544008 28777094

[20] A. Derardja , M. Pretzler , I. Kampatsikas , M. Barkat , A. Rompel , J. Agric. Food Chem. 2017, 65, 8203–8212. 28812349 10.1021/acs.jafc.7b03210 PMC5609118

[21] E. Krissinel , K. Henrick , J. Mol. Biol. 2007, 372, 774–797. 17681537 10.1016/j.jmb.2007.05.022

[22] F. Zekiri , C. Molitor , S. G. Mauracher , C. Michael , R. L. Mayer , C. Gerner , A. Rompel , Phytochemistry 2014, 101, 5–15. 24613318 10.1016/j.phytochem.2014.02.010 PMC3989047

[23] F. Zekiri , A. Bijelic , C. Molitor , A. Rompel , Acta Crystallogr. Sect. F 2014, 70, 832–834. 10.1107/S2053230X1400884X PMC4051548 24915104

[24] A. Bijelic , M. Pretzler , C. Molitor , F. Zekiri , A. Rompel , Angew. Chem. Int. Ed. 2015, 54, 14677–14680; 10.1002/anie.201506994 PMC4678486 26473311

[25] T. Klabunde , C. Eicken , J. C. Sacchettini , B. Krebs , Nat. Struct. Mol. Biol. 1998, 5, 1084–1090. 10.1038/4193 9846879

[26] C. Kaintz , C. Molitor , J. Thill , I. Kampatsikas , C. Michael , H. Halbwirth , A. Rompel , FEBS Lett. 2014, 588, 3417–3426. 25109778 10.1016/j.febslet.2014.07.034 PMC4158910

[27] C. Molitor , S. G. Mauracher , A. Rompel , Acta Crystallogr. Sect. F 2015, 71, 746–751. 10.1107/S2053230X15007542 PMC4461341 26057806

[28] B. Webb , A. Sali , Curr. Protoc. Bioinf. 2016, 54, 56.1–5.6.37. 10.1002/cpbi.3 PMC5031415 27322406

[29] K. Arnold , L. Bordoli , J. Kopp , T. Schwede , Bioinformatics 2006, 22, 195–201. 16301204 10.1093/bioinformatics/bti770

