# Supplementary material for: A Peptide‐Induced Self‐Cleavage Reaction Initiates the Activation of Tyrosinase
Source: Angew Chem Int Ed Engl. 2019 Apr 17;58(22):7475–9. doi: 10.1002/anie.201901332 (PMC6563526; doi:10.1002/anie.201901332)
Supplement: Supplementary file 1 — Supplementary [file ANIE-58-7475-s001.pdf]

Angewandte Chemie
Eine Zeitschrift der Gesellschaft Deutscher Chemiker

Supporting Information

A Peptide-Induced Self-Cleavage Reaction Initiates the Activation of Tyrosinase

*Ioannis Kampatsikas, Aleksandar Bijelic, Matthias Pretzler, and Annette Rompel**

anie_201901332_sm_miscellaneous_information.pdf

Table of Contents

1. Supplementary Material and Methods 2

2. Supplementary Notes 5

3. Supplementary Figures 8

4. Supplementary Tables 27

5. References 30

1. Supplementary Materials and Methods

Plasmid preparation and heterologous expression of pro-*Md*PPO1 in *E. coli*. The pro-form of *Md*PPO1 was heterologously expressed and purified as described elsewhere.[1]

Plasmid preparation and heterologous expression of the C_{sole}-domain in *E. coli*. The plasmid of *Md*PPO1 in the pGEX-6P-1 vector was used as template to create the C-terminal domain construct in the pGEX-6P-1 vector for heterologous expression in *E. coli*. Primers have been designed (Table S2) and site direct mutagenesis (Q5® Site-Directed Mutagenesis Kit from New England Biolabs) was used in order to delete the active domain from the plasmid and bring the C-terminal domain in frame with the glutathione-S-transferase (GST) tag. The fusion gene with the C-terminal domain (GST-C_{sole}) was efficiently overexpressed using the synthetic *tac* promoter of the pGEX-6P-1 vector. The expression and purification of the C_{sole}-domain followed the same protocol as the one described previously for pro-*Md*PPO1.[1] C_{sole}-domain was stored in 50 mM Tris-HCl pH 7.5 and 200 mM NaCl and was used for crystallographic and biochemical studies.

Design of the mutants and heterologous expression in *E. coli*. The plasmid of *Md*PPO1, *Md*PPO2 and *Cg*AUS1 in the pGEX-6P-1 vector were used as templates for the construction of the mutants (Table S1) in pGEX-6P-1 for heterologous expression in *E. coli*. Primers have been designed (Table S2) and site direct mutagenesis (Q5® Site-Directed Mutagenesis Kit) was used. The mutant fusion genes were efficiently overexpressed using the synthetic *tac* promoter of the pGEX-6P-1 vector. The heterologous expression of all the mutants followed the same protocol described previously for pro-*Md*PPO1.[1]

SDS-PAGE analysis and protein concentration determination. The quality of heterologous expression and the purity of the recombinant proteins were analyzed *via* SDS-PAGE using 13% acrylamide gels.[2] The samples were mixed with half a volume of loading buffer (250 mM Tris-HCl pH 6.8, 3% SDS, 20% glycerol, 0.02% bromophenol blue and 7.5% (v/v) β-mercaptoethanol) and were denatured at high temperature (99 °C) for 5 minutes. Afterwards the samples were loaded on the SDS-PAGE gel and run in a Mini-PROTEAN Tetra Cell System (Biorad, Vienna, Austria). Precision Plus Protein Dual Color Standard (Biorad) was used as protein-marker. The separated proteins were visualized by Coomassie staining (0,02% Coomassie brilliant blue G-250, 5 % aluminium sulfate, 10 % ethanol and 2% orthophosphoric acid). Protein concentrations were determined according to the Lambert-Beer law and their absorption at 280 nm using the extinction coefficient provided by ExPASy ProtParam.[3,4]

Molecular mass determination by ESI-QTOF-MS and ESI LTQ Orbitrap Velos. Electrospray Ionization Mass Spectrometry (ESI-MS) was performed on a nano electrospray ionization - quadrupol and time-of-flight mass spectrometer (ESI-QTOF-MS, MaXis 4G UHR-TOF, Bruker) with a mass range of 50 - 20000 m/z. Pure pro-*Md*PPO1 (already self-cleaved into the active form and the $C_{cleaved}$-domain), as well as mutation-1, mutation-2 and the rest of the mutants of (Table S1) (already self-cleaved into the active form and the C-terminal domain) were used at a concentration of 10 g/l. The buffer was exchanged to 5 mM ammonium acetate (pH 7.0) and the protein solutions were diluted to 1 % (v/v) in 2 % acetonitrile and 1 ‰ formic acid before being applied to the mass spectrometer. Mass determination of *Md*PPO2(+) and *Cg*AUS1(+) (already self-cleaved into the active form and the C-terminal domain) was performed by an ESI - LTQ - Orbitrap Velos (Thermo Fisher Scientific Bremen, Germany) with a mass range of 200 - 4000 m/z and a mass accuracy

close to 3 ppm with external calibration. Prior to MS *Md*PPO2(+) solution was ultra-filtrated by centrifugation and the buffer was exchanged to 5 mM ammonium acetate (pH 7.0) and the protein solution was diluted 100 times in a mixture of 80 % (v/v) acetonitrile and 0.1 % (v/v) formic acid.

Self-cleaving of *Md*PPO1 at different temperatures. Pro-*Md*PPO1 was investigated at different temperatures in order to examine the influence of the temperature on self-cleaving. The enzyme was incubated at the following temperatures: 4, 20, 25, 37 and 65 °C. Samples were tested after different incubation times *via* SDS-PAGE (Figure S6).

Self-cleaving of *Md*PPO1 at different pH values. Pro-*Md*PPO1 was investigated at different pH values in order to examine the influence of pH on self-cleaving. 50 mM sodium citrate buffer was used to analyze the pH-range between pH 2.0 and 6.0 in steps of 1 pH units, whereas 50 mM Tris-HCl buffer was used to investigate the process in the range between pH 7.0 to 9.0 in steps of 1 pH units. Samples were stored at 4 °C and tested after different incubation times *via* SDS-PAGE (Figure S7).

Influence of protease inhibitors on the self-cleaving of pro-*Md*PPO1. Pro-*Md*PPO1 was incubated with a range of protease inhibitors to investigate their influence on the self-cleaving process. For this purpose 300 μg of pro-*Md*PPO1 (~265 μM) was incubated with 2 mM phenylmethylsulfonyl fluoride (Karl Roth, Karlsruhe, Germany), 2 mM benzamidine hydrochloride (Sigma-Aldrich, Vienna, Austria), 10 mM EDTA (Sigma-Aldrich) and 0.1 - 50 μM pepstatin A (Sigma-Aldrich), respectively. In addition, the commercially available mixture of protease inhibitors SigmaFAST (Sigma-Aldrich) was examined. The final assay mixture contained 300 μg of *Md*PPO1, 2 mM AEBSF–[4-(2-Aminoethyl)benzenesulfonyl fluoride hydrochloride], 130 μM bestatin hydrochloride, 1 μM leupeptin, 14 μM E-64 -[N-(trans-Epoxysuccinyl)-L-leucine 4-guanidinobutylamide], 0.2 μM aprotinin, 10 μM pepstatin A and 1 μM phosphoramidon disodium salt. All incubation trials were carried out in the storage buffer (50 mM Tris-HCl pH 7.5 and 200 mM NaCl) at 4 °C (Figure S8 and S9).

Crystallization. Crystallization of pro-*Md*PPO1 was performed as reported elsewhere.[5] To obtain crystals of the $C_{cleaved}$-domain, a solution containing the pro-enzyme was prepared and was stored more than 30 days at 4 °C to let the self-cleaving process take place. The self-cleaved sample was used for initial screening, which was performed manually by applying the hanging-drop vapor-diffusion technique using 15 well EasyXtal plates (Qiagen, Hilden, Germany). The screening procedure yielded some promising hits (small crystals). The initial hits were optimized and high quality crystals of the $C_{cleaved}$-domain were finally grown at 20 °C by mixing 1 μl protein solution (15 mg ml^{-1}) with 1 μl of the reservoir solution (100 mM Tris-HCl pH 8.25, 13 % PEG8000). Crystals usually appeared after 5 - 10 d. The same conditions were used to crystallize the C_{sole}-domain.

Data collection, structure solution and refinement. Crystal harvesting and data collection of pro-*Md*PPO1 was performed as described elsewhere.[5] The crystals of the $C_{cleaved}$- and C_{sole}-domain were quickly plunged into cryo-protectant solution consisting of 100 mM Tris-HCl at pH 8.25, 20% PEG 8000 and 25% PEG 1500 and were afterwards mounted in nylon loops and immediately flash-cooled in liquid nitrogen. Data collection of the $C_{cleaved}$-domain was carried out at 100 K on beamline ID-30A-1, at the ESRF, Grenoble, France, whereas data of the C_{sole}-domain were collected on beamline ID-23-1, at the same synchrotron facility. Data collection statistics are summarized in Table S3. $C_{cleaved}$-domain crystals diffracted X-rays to a maximum resolution of 1.35 Å, whereas those of the C_{sole}-domain reached

resolutions up to 1.05 Å. All crystals belonged to space group $P2_12_12_1$ exhibiting the following cell parameters: a = 45.23 Å, b = 50.14 Å, c = 50.72 Å with α = 90°, β = 90° and γ = 90° for the $C_{cleaved}$-domain and a = 45.13 Å, b = 50.10 Å, c = 50.54 Å with α = 90°, β = 90° and γ = 90° for the C_{sole}-domain. Data sets of the C-terminal domain were processed with XDS.[6]

The structure of pro-*Md*PPO1 (including the N-terminal domain) was solved by molecular replacement (MR).[5] To find an appropriate model for the (MR) step, a BLAST search[7] was performed using the sequence of pro-*Md*PPO1 as template. The BLAST results provided several promising MR-models, where the model with the highest coverage (0.97 according to BLAST) was the structure of aurone synthase from *Coreopsis grandiflora* (PDB entry 4Z11, sequence identity of 43.0%),[8,9] and the model with the highest sequence identity was the structure of tyrosinase from *Juglans regia* (PDB entry 5CE9, sequence identity of 66.6%).[10,11] Initial MR-runs with both potential models using phenix.phaser from the PHENIX suite[12] revealed that the tyrosinase structure was more suitable as MR-model owing to higher MR-scores in comparison to those obtained with the structure of aurone synthase. The final MR-model was obtained by modifying the tyrosinase structure with the sequence of pro-*Md*PPO1 using the modelling software MODELLER.[13] After initial phases were derived, AutoBuild[14] was used to build the initial *Md*PPO1 model, which was then refined until convergence using phenix.refine.[12] The quality of the final model was verified and evaluated by the MolProbity server and deposited in the PDB under the entry 6ELS. The structures of the C-terminal domains were solved as described above using the C-terminal domain of the solved pro-*Md*PPO1 as MR model .The final structures of the $C_{cleaved}$- and C_{sole}-domain were deposited in the PDB and may be retrieved from the entries 6ELT and 6ELV, respectively.

2. Supplementary Notes

The overall structure of pro-*Md*PPO1. The active site region of *Md*PPO1 consists of a binuclear copper center (CuA and CuB), where each copper ion is coordinated by three histidine residues (CuA by His86, His107 and His116 and CuB by His238, His242 and His272), and is wrapped by a four-α-helical bundle. The structure lacks two highly conserved disulfide bonds (Cys11-Cys26 and Cys25-Cys87) most likely due to *Md*PPO1 expression in *E. coli*, an organism which lacks the appropriate machinery for cystine formation. Moreover, despite the high resolution, the structure of pro-*Md*PPO1 suffers from some structural imperfections due to missing electron density, namely at the N-terminus (the sequence of the structure starts with Lys32) and three gaps within loop regions (His86-Gln105, Ala349-Val359 and His453-Lys457). The largest gap from His86 to Gln105 is also the most critical one as it affects a loop connecting two α-helices within the active site forming α-helical bundle. However, some electron density for the CuA-coordinating His86 was found enabling the structural completion of the active site, and in addition, the sulfur atom of the adjacent Cys90 was detected indicating that the thioether bridge, which is conserved among structurally known plant PPOs, is at least partially formed within the structure. The lack of the disulfide bonds (Cys11-Cys26 and Cys25-Cys87) could also explain the missing initial part in the structure as they highly contribute to the structural stability of the N-terminus by anchoring the N-terminal tail to the main core of the enzyme. Thus, the here reported structure contains a very loose N-terminus, which does not produce detectable electron density for the first 31 residues due to its high flexibility. In other plant PPO structures the N-terminus embraces one of the four α-helices of the tetrahelical bundle representing some kind of specific fold. As the N-terminus of *Md*PPO1 is highly flexible owning due to the lack of the two disulfide bonds, the above described structural region is partially disordered in the *Md*PPO1 structure (Figure S17).

Mutagenesis studies on *Md*PPO1 and the relocation of the cleavage position. In order to gain additional insights into the cleavage reaction the cleavage site Ser366-Ser367-Ser368-Lys369-Val370 was mutated to Ile367-Asp368-Gly369-Arg370 (mutant-1), which corresponds to the recognition sequence of the serine protease Factor Xa. Thus, mutant-1 was not only designed to test its influence on the self-cleaving process but also to enable cleavage by the use of the external protease Factor Xa. Mutant-1 was successfully cleaved by Factor Xa protease yielding a similar pre-active state as in the case of *Md*PPO1 undergoing self-cleaving. ESI-MS analysis of the fragments obtained upon Factor Xa cleavage revealed two new cleavage sites for the mutated enzyme, which in comparison to the wild-type are shifted by 11 amino acids towards the N-terminus and consist of the sequence Lys355-Lys356-Leu357 (Figure S10). Moreover, in a second mutant (mutation-2) the two proteolytic sites (Ser366-Ser367-Ser368-Lys369-Val370) and (Lys355-Lys356-Leu357) were mutated (Table S1). However, a third proteolytic region was appeared (His361-Ala362-Ala363) in between the two previous (Figure S11). Mass spectrometry results (Figures S3, S10 and S11) show that the cleavage site of *Md*PPO1 is not characterized by one single peptide bond but rather by a cleavage sequence consisting of four contiguous peptide bonds (Ser366-Ser367-Ser368-Lys369-Val370) for the wild type, two contiguous peptide bonds (Lys355-Lys356-Leu357) for the mutation-1 and two contiguous peptide bonds (His361-Ala362-Ala363) for the mutation-2 exhibiting very high specificity as evidenced by mass spectrometry and the sequence of the $C_{cleaved}$-domain crystal structure. Moreover, several mutants were designed in *Md*PPO1 in order to stop the self-cleaving

reaction however without success the mutant which stops the self-cleaving reaction was the deletion of the loop Lys355-Val371 and described in Table S1.

Pro-*Md*PPO1 activation requires two steps. Despite undergoing self-cleaving, activity tests on the cleaved pro-*Md*PPO1 revealed that the enzyme retained its latency. This observation implies that the proteolytic cleavage within the sequence Ser366-Ser367-Ser368-Lys369-Val370 alone is not sufficient to activate the pro-enzyme. Visual inspection and analysis of the interface between the C-terminal and main-domain by PISA analysis[15] revealed strong interactions between the two domains. 33 H-bonds and 13 salt-bridges were detected by PISA analysis and therefore it is suggested that even after the proteolytic reaction, the domains remain attached to each other due to strong electrostatic interface interactions, thereby preserving the enzyme's latency. Thus, self-cleaving of the pro-enzyme converts the enzyme into a stable, pre-active stage, this requires further actions to become fully enzymatically active. Self-cleaved enzyme was *in vitro* activated by applying various salt concentrations (1 M NaCl, 0.5 M KCl, 0.15 M $MgCl_2$ or 0.15 M $CaCl_2$), which putatively disrupt the strong electrostatic inter-domain interactions leading to the spatial separation of the two domains. The same effect was also achieved using the activator SDS (3 mM). However, this *in vitro* activation process might not reflect the *in vivo* maturation process but it is suggested that the enzyme is transformed to the here observed pre-active stage at the same point during the *in vivo* maturation. The C-terminal domain of *Md*PPO1 could be membrane associated as it has been reported for other PPOs.[16–18] Thus, the spatial separation of the domains might *in vivo* be triggered by the fact that C-terminal domain remains attached to a membrane facilitating the departure of the main domain.

Experiments to elucidate the mode of the self-cleaving reaction. A series of experiments were conducted to reveal the mechanism of the self-cleaving reaction. The initial consideration was that the C-terminal domain harbors the cleaving moiety. This was supported by the fact that incubation of the pro-*Md*PPO1 together with different amounts of separated C-terminal domain (C_{sole}-domain) accelerated the self-cleaving reaction. Structural inspection of the C-terminal domain did, however, not reveal an obvious cleaving element. Thus, the structure of the C-terminal domain ($C_{cleaved}$, C_{sole} and the C-terminal domain of the pro-form) was submitted to the Dali Server, a network for (3D) structural protein comparison, to find similar structures or structural elements in other enzymes that might have proteolytic activity. However, the best hits (besides other PPOs) were the nephrin-binding-domains of several nephrin-receptors[19]. No proteolytic enzyme was within the top hits.

In addition, several proteases inhibitors were incubated with pro-*Md*PPO1 to inhibit the self-cleaving process. The serine protease inhibitors 2 mM phenylmethylsulfonyl fluoride and 2 mM benzamidine hydrochloride did slightly attenuate the self-cleaving process, a serine protease-like mechanism was assumed. Analysis of the sequence in the proximity of the cleavage site revealed the presence of a number of serine residues that could act as the decisive nucleophile. However, when the environment of each serine was examined, no acid and base were found that could form a catalytic triad to carry out the reaction. Only the positioning of S435 resembled to some extent that found in the catalytic moiety of thrombin. Furthermore, there are reports on serine proteases that are able to perform the cleavage reaction in a "serine-only" configuration, that is, only the serine residue is required for the self-cleaving reaction of some proteins.[20,21] Therefore, Ser385 and Ser435 were mutated to Gly in different mutants to inhibit the putative serine protease-like mechanism. However, self-cleaving in the resulting mutant was only delayed but not inhibited completely discarding a

serine protease-like reaction as mode of action for the here observed self-cleaving (Table S1).

Since EDTA did also attenuate the self-cleaving process, a metalloprotease-like reaction was also shortly assumed. This assumption was further strengthening by the fact that the separated C-terminal domain possesses a (strong) metal binding site and might thus be the decisive element. Incubation of different metals, 1 mM of (Mg^{2+}, Mn^{2+}, Co^{2+}, Ni^{2+}, Zn^{2+}, Cu^{2+} and Ca^{2+}) with pro-*Md*PPO1 did not show metal-dependent differences or any effect at all. In addition, the calcium binding residues Asp429, Asp431 and Asp479 were mutated to Gly or deleted completely in order to block the metal binding site. However, the resulting mutations did not inhibit the self-cleaving reaction but led to the opposite effect as self-cleaving was accelerated (Table S1). Thus, a metalloprotease-like or in general metal-based reaction was also excluded.

Structural comparison between the C-terminal domains (C-terminal domain attached in pro-*Md*PPO1, $C_{cleaved}$ and C_{sole}-domain). Structural comparison between the C-terminal domain of the pro-enzyme, the $C_{cleaved}$- and C_{sole}-domains, revealed that the two free C-terminal domains ($C_{cleaved}$ and C_{sole}) exhibit the exact same structure (Cα-RMSD = 0.051 Å, 872 matched atoms), whereas the structure of the C-terminal domain of the pro-enzyme does slightly deviate from the free C-terminal domains (Cα-RMSD of 0.494 Å, 562 matched atoms). All C-terminal domains exhibit the typical β-sandwich 'jelly roll'-like fold consisting of in total seven β-strands (Figure S18), however, the position of one large loop (Asn428-Glu441) differs significantly. In the structures of the separate $C_{cleaved}$- or C_{sole}-domain, this loop moved towards another loop (Glu473-Asp480) in order to form a calcium binding site that stabilizes the C-terminal domain (Figure S19). The bound metal was identified as Ca^{+2} based on the composition of the used expression media and buffers, the interacting amino acids, the binding geometry and the presence of the anomalous signal (Figure S20). The Ca^{+2} ion is coordinated by eight O-donor ligands, three aspartate residues (Asp429, Asp431 and Asp479), whereby two of these aspartates (Asp429 and Asp479) act as bidentate ligands and three oxygens arising most likely from water molecules (Figure S21). The calcium binding site is missing in the structure of pro-*Md*PPO1 as the two loops forming this site are kept apart by the linker region, which connects the active and C-terminal domain.

3. Supplementary Figures

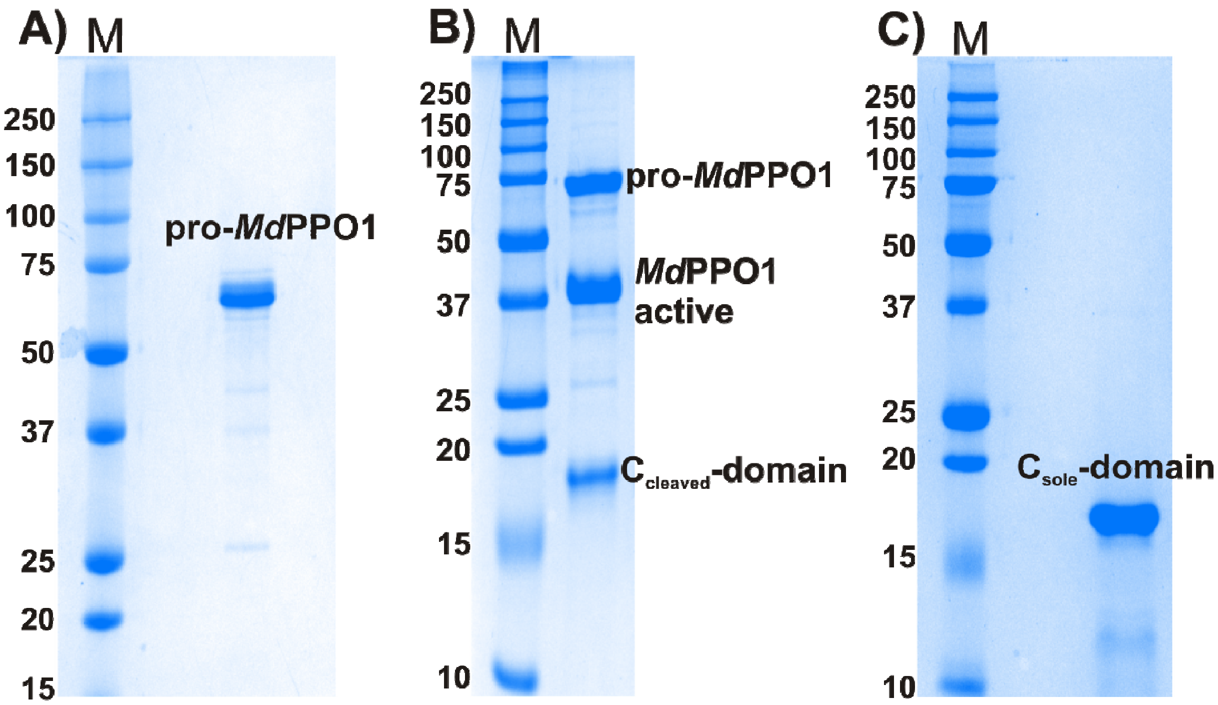

Figure S1. SDS PAGE (under reducing conditions) showing the purity of the investigated proteins. (A) pro-*Md*PPO1, (B) pro-*Md*PPO1 upon partial self-cleaving resulting in the appearance of the active enzyme and the C-terminal domain ($C_{cleaved}$-domain), (C) recombinantly produced separated C-terminal domain (C_{sole}-domain). M indicates the marker (molecular masses are given in kDa).

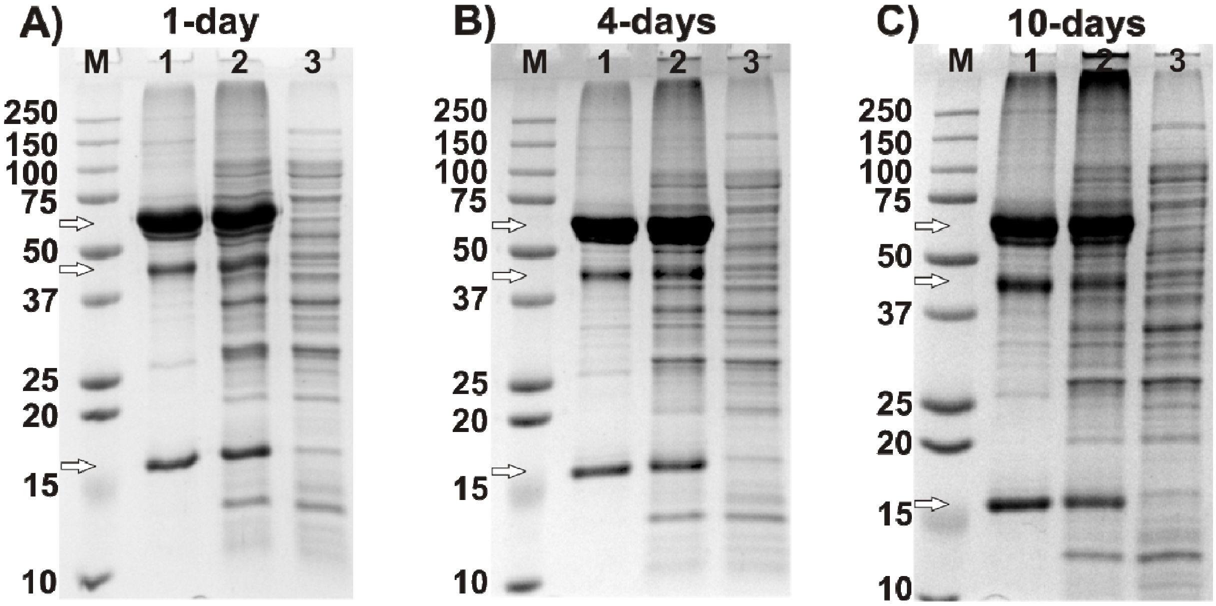

Figure S2. SDS PAGE (under reducing conditions) showing the self-cleaving process of *Md*PPO1 during time in comparison to the self-cleaving process in a mixture with lysate of *E. coli*. The gels in A-C) show samples reacting for the time interval indicated above the respective gel. Lane 1) 10 µg of *Md*PPO1, lane 2) 10 µg of *Md*PPO1 mixing with 50 µg of *E. coli* lysate, lane 3) 50 µg of *E. coli* lysate. The arrows indicate the positions of the pro-enzyme (top), the active enzyme (middle) and the C-terminal domain (bottom).

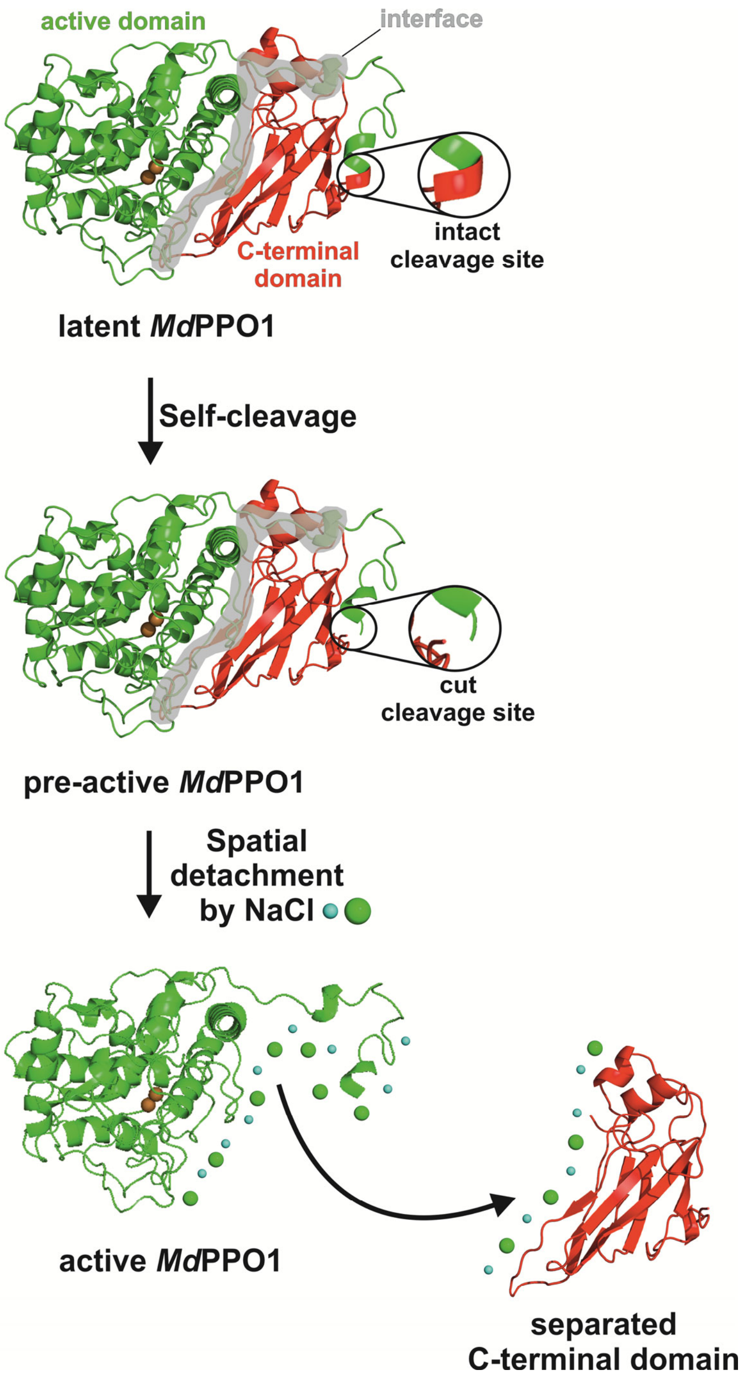

Figure S3. *Md*PPO1 activation requires two steps. *Md*PPO1 reveals a self-cleaving reaction within the sequence Ser366-Ser367-Ser368-Lys369-Val370, although, it is not sufficient to activate the pro-enzyme. The two domains C- and N-terminal reveal strong interactions between them and remain intact. 33 H-bonds and 13 salt-bridges were detected by PISA analysis, therefore, cleaved *Md*PPO1 requires further actions to become fully enzymatically active. Self-cleaved *Md*PPO1 was *in vitro* activated by applying various salt concentrations (1 M NaCl, 0.5 M KCl, 0.15 M $MgCl_2$ or 0.15 M $CaCl_2$), which putatively disrupt the strong electrostatic inter-domain interactions leading to the spatial separation of the two domains, as represented here with the use of NaCl.

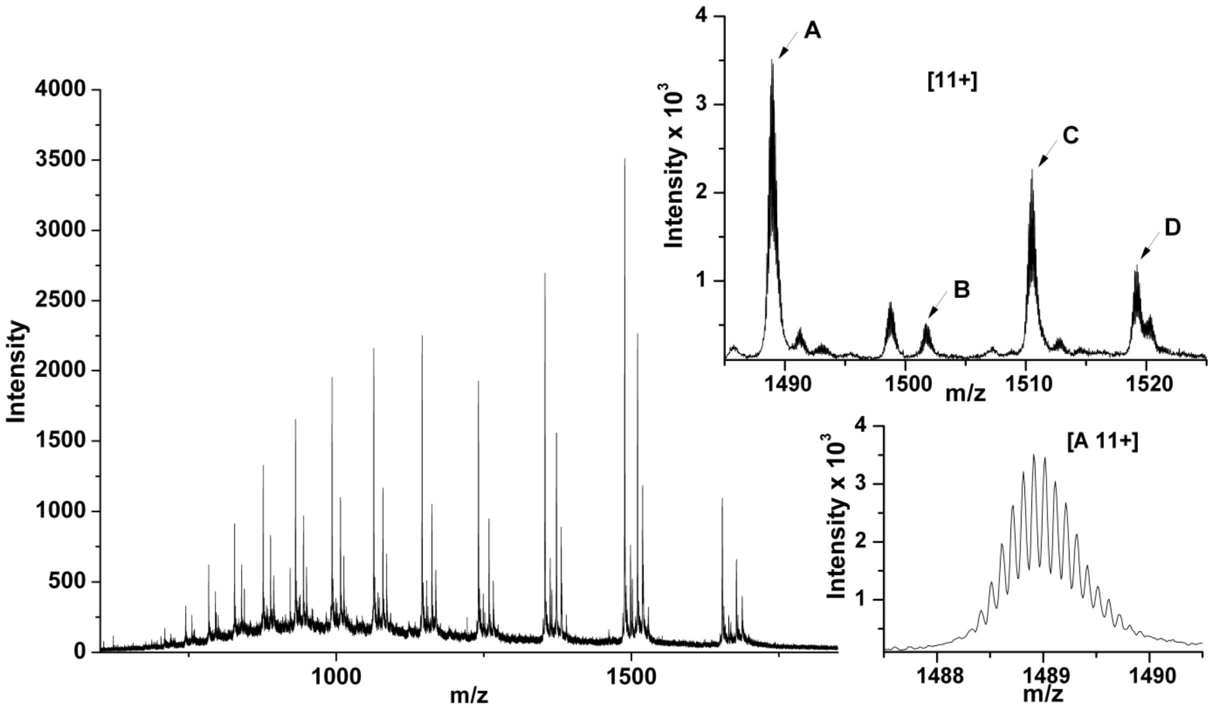

Figure S4. Positive mode ESI-QTOF mass spectra of the $C_{cleaved}$-domain obtained by self-cleaving from pro-*Md*PPO1. During and after self-cleavage the sample solution represents a mixture of pro-, active *Md*PPO1 as well as of the $C_{cleaved}$-domain (Figure S1). However, the $C_{cleaved}$-domain was ionized best and led to the here presented high-resolution spectra. The inset shows that four different C-terminal species (A-D) were found and the second inset of the charge state [11+] shows that isotopic resolution was achieved. The four different species indicate self-cleaving at four different sites within the sequence (Ser366-Ser367-Ser368-Lys369-Val370). Species A corresponds to the fragment obtained upon cleavage between Lys369-Val370, species B to cleavage between Ser368-Lys369, species C to cleavage between Ser367-Ser368 and species D to cleavage between Ser366-Ser367.

Species	M calculated Da	M (measured) Da	Δ/Da
A	14879.42	14879.87	-0.45
B	15008.65	15008.05	+0.61
C	15095.02	15095.12	-0.11
D	15181.57	15182.20	-0.63

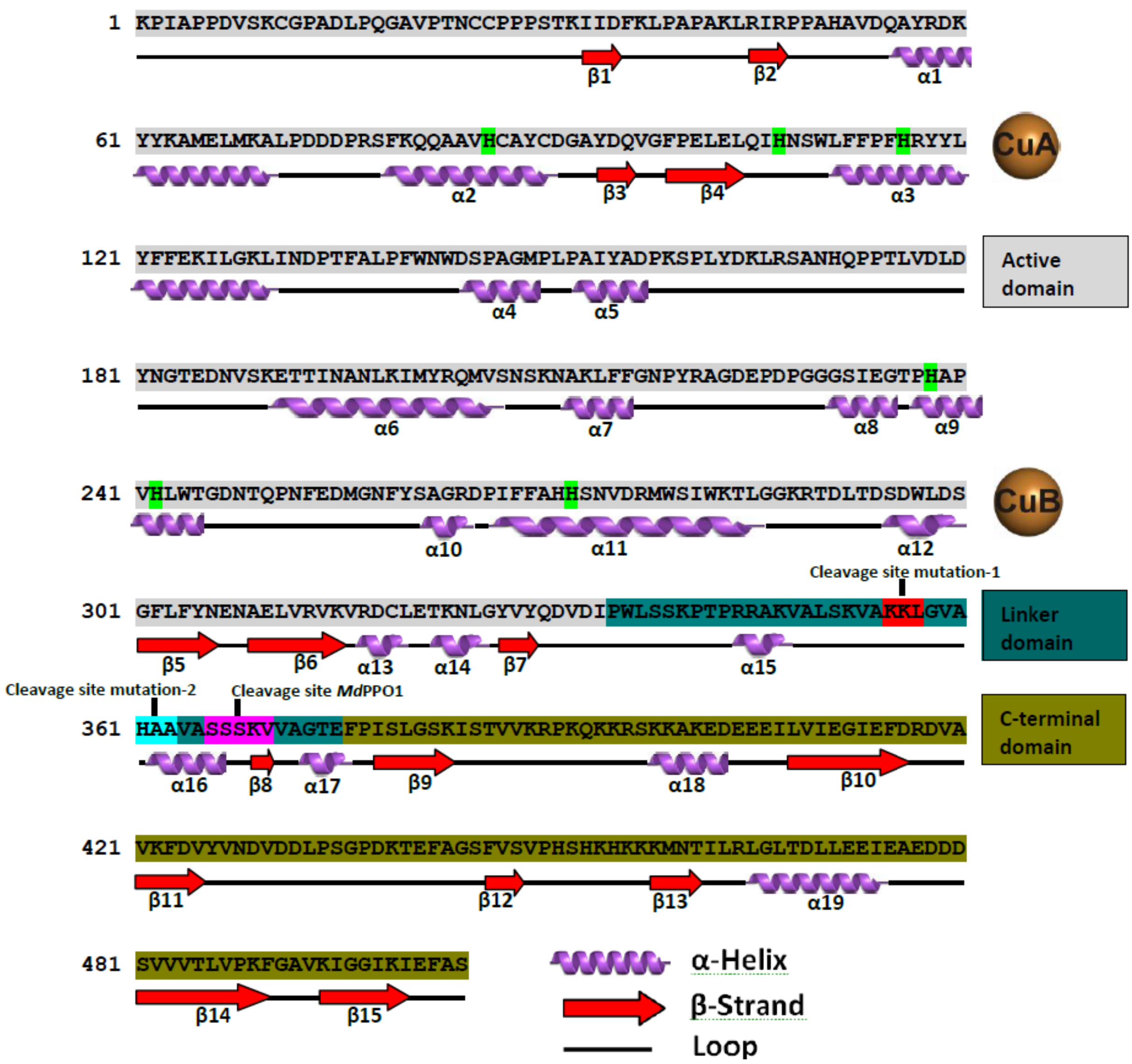

Figure S5. Primary and secondary structure of pro-*Md*PPO1. The following regions are highlighted: active domain (grey amino acids), linker domain (blue-green) and the C-terminal domain (olive), dicopper center (CuA and CuB) and the conserved copper-coordinating histidines (green). Moreover, the cleavage sites of the wild type *Md*PPO1 (Ser366-Ser367-Ser368-Lys369-Val370, pink), the mutation-1 (Lys355-Lys356-Leu357, red) and the mutation-2 (His361-Ala362-Ala363, cyan) are marked.

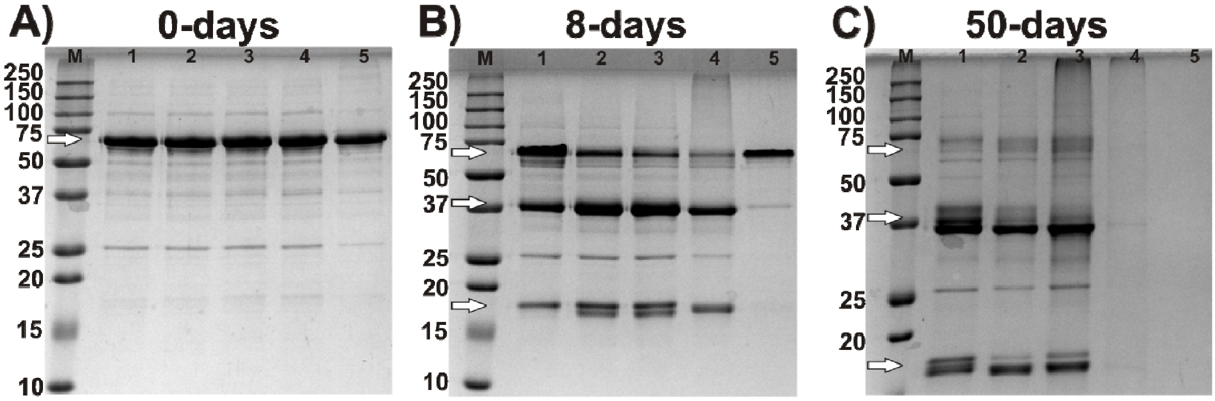

Figure S6. SDS-PAGE of *Md*PPO1 at different temperatures and time intervals. In every lane 5 µg of pro-*Md*PPO1 were applied. The gels in A-C) show samples reacting for the time interval indicated above the respective gel. Lane 1) 300 µg of *Md*PPO1 at 4 °C, lane 2) 300 µg of *Md*PPO1 at 20 °C, lane 3) 300 µg of *Md*PPO1 at 25 °C, lane 4) 300 µg of *Md*PPO1 at 37 °C and lane 5) 300 µg of *Md*PPO1 at 65 °C. M indicates the molecular marker (molecular masses in kDa). Self-cleavage of *Md*PPO1 was fastest at 37 °C. After 8 days the pro-*Md*PPO1 has been cleaved completely at 37 °C and almost completely at 20 °C and 25 °C. At 4 °C the proteolysis proceeds slower, whereas at 65 °C the self-cleavage is negligible. After 50 days the protein was completely cleaved at 4 °C, 20 °C and 25 °C but precipitated at 37 °C and 65 °C. The arrows indicate the positions of the pro-enzyme (top), the active enzyme (middle) and the C-terminal domain (bottom).

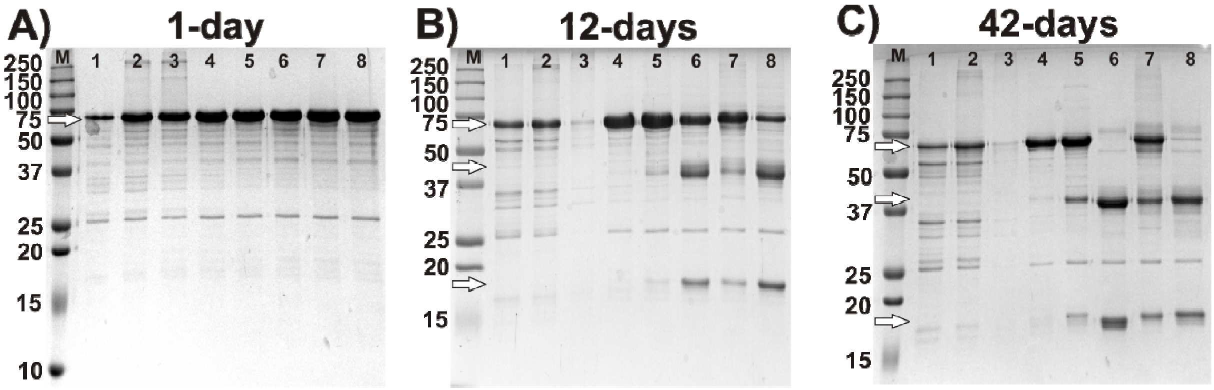

Figure S7. SDS-PAGE of *Md*PPO1 at different pH values and time intervals. In every lane 5 µg of the pro-*Md*PPO1 were applied. The gels in A)-C) show samples reacting for the time interval indicated above the respective gel. Lane 1) 300 µg of *Md*PPO1 at pH 2, lane 2) 300 µg of *Md*PPO1 at pH 3, lane 3) 300 µg of *Md*PPO1 at pH 4, lane 4) 300 µg of *Md*PPO1 at pH 5, lane 5) 300 µg of *Md*PPO1 at pH 6, lane 6) 300 µg of *Md*PPO1 at pH 7, lane 7) 300 µg of *Md*PPO1 at pH 8 and lane 8) 300 µg of *Md*PPO1 at pH 9. M indicates the molecular marker (molecular masses in kDa). *Md*PPO1 shows a clear pH-dependent behavior. At pH 2 and 3, *Md*PPO1 is hardly stable and therefore the self-cleavage process could not be tracked appropriately. After 6 days *Md*PPO1 precipitates at pH 4. At pH 5 it seems that the self-cleavage does not take place at all as the band of the enzyme does not change even after 42 days. At pH 6 the self-cleavage is very slow as the active enzyme and the C-terminal domain start to be obvious only after 42 days. The pH values of 7 and 9 seem to represent the optimal pH for the self-cleavage process as the active enzyme appears between 6 to 13 days. The enzyme is completely cleaved after 42 to 57 days at these pH values. At pH 8 the reaction shows an interesting delay and seems to be similar to the reaction rate at pH 6. The arrows indicate the positions of the pro-enzyme (top), the active enzyme (middle) and the C-terminal domain (bottom).

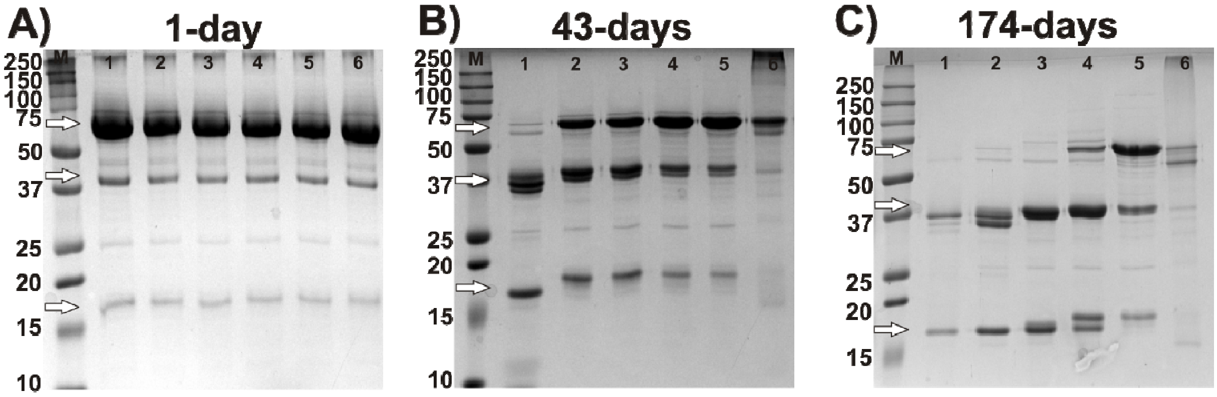

Figure S8. SDS-PAGE of *Md*PPO1 incubated with serine protease inhibitors phenylmethylsulfonyl fluoride (PMSF) and benzamidine the metalloprotease inhibitor (EDTA) and a commercial protease inhibitor mixture (SigmaFAST) for different time intervals. In every lane 5 µg of the pro-*Md*PPO1 were applied. The gels in A)-C) show samples reacting for the interval of time. Lane 1) 300 µg of *Md*PPO1, lane 2) 300 µg of *Md*PPO1 with 2 mM PMSF, lane 3) 300 µg of *Md*PPO1 with 2 mM benzamidine, lane 4) 300 µg of *Md*PPO1 with 10 mM EDTA, lane 5) 300 µg of *Md*PPO1 with 2 mM PMSF, 2 mM benzamidine and 10 mM EDTA and lane 6) 300 µg of *Md*PPO1 with 10 µl SigmaFAST protease inhibitor mix (1 tablet in 5 ml). The self-cleavage reaction of *Md*PPO1 was remarkably inhibited by both serine protease inhibitors (PMSF and benzamidine) but also by the metalloprotease inhibitor (EDTA). The proteolytic reaction in the samples containing PMSF and benzamidine finished only after 80 days in comparison to the natural reaction which took only 14 to 17 days. *Md*PPO1 very slowly cleaved in the solution of SigmaFAST protease inhibitor mix while, the cleaved product precipitated. However, the herein investigated protease inhibitors were not able to stop completely the self-cleaving reaction. The arrows indicate the positions of the pro-enzyme (top), the active enzyme (middle) and the C-terminal domain (bottom).

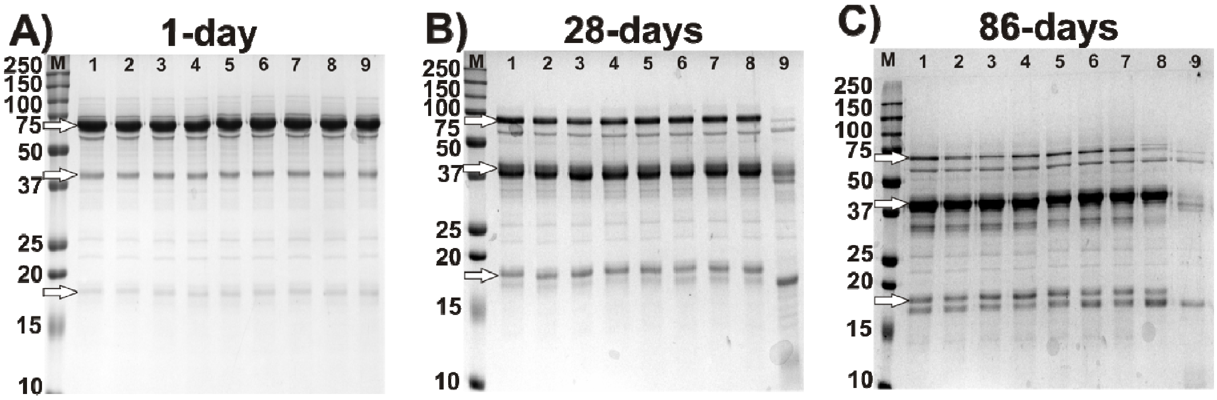

Figure S9. SDS-PAGE of *Md*PPO1 incubated with the aspartyl protease inhibitor pepstatin A at different time intervals. In every lane 5 µg of pro-*Md*PPO1 were applied. The gels in A)-C) show samples reacting for the time interval indicated above the respective gel. Lane 1*)* 300 µg of *Md*PPO1 with 0 mM pepstatin A, lane 2) 300 µg of *Md*PPO1 with 0.1 µM pepstatin A, lane 3) 300 µg of *Md*PPO1 with 0.25 µM pepstatin A, lane 4) 300 µg of *Md*PPO1 with 0.5 µM pepstatin A, lane 5) 300 µg of *Md*PPO1 with 1 µM pepstatin A, lane 6) 300 µg of *Md*PPO1 with 5 µM pepstatin A, lane 7) 300 µg of *Md*PPO1 with 10 µM pepstatin A, lane 8) 300 µg of *Md*PPO1 with 20 µM pepstatin A and lane 9) 300 µg of *Md*PPO1 with 50 µM pepstatin A. The aspartyl protease inhibitor pepstatin A seems to have no influence on the self-cleavage. All samples reacted similarly without the self-cleavage being inhibited by pepstatin A. The arrows indicate the positions of the pro-enzyme (top), the active enzyme (middle) and the C-terminal domain (bottom).

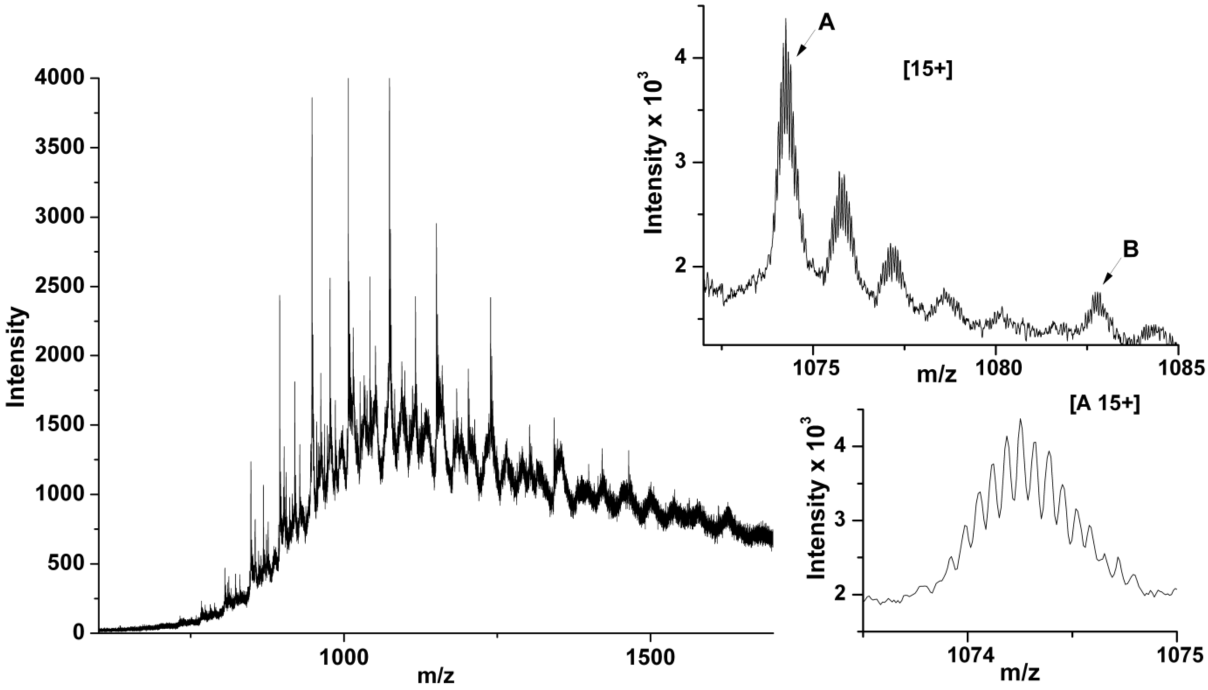

Figure S10. Positive mode ESI-QTOF mass spectra of the self-cleaved $C_{cleaved}$-domain from mutation-1. During and after self-cleaving the sample solution represents a mixture of pro- and active *Md*PPO1 as well as the free C-terminal domain. However, the C-terminal domain was ionized best and led to the here presented high-resolution spectra. The inset shows that two different species (A-B) were found and the second inset of the charge state [15+] shows that isotopic resolution was achieved. The two different species indicate self-cleaving at two different sites within the sequence (Lys355-Lys356-Leu357). Species A corresponds to the fragment obtained upon cleavage between Lys356-Leu357, species B to cleavage between Lys355-Lys356.

Species	M calculated Da	M (measured) Da	Δ/Da
A	16099.22	16099.23	+0.01
B	16227.40	16227.64	+0.25

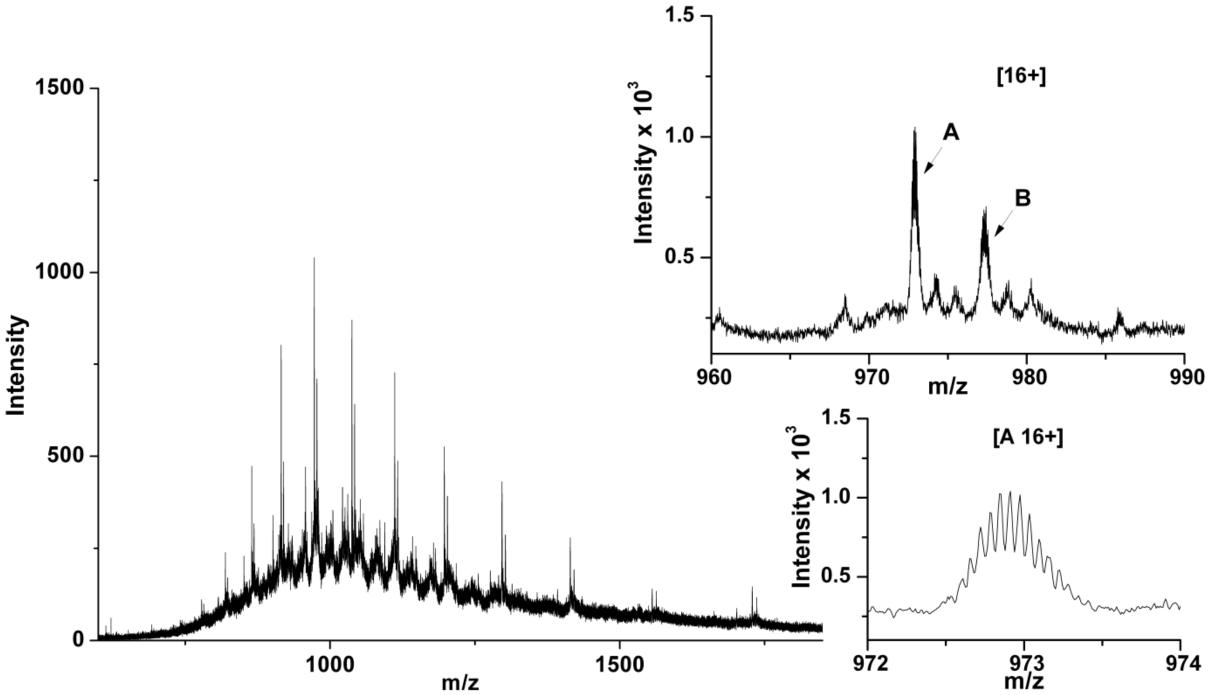

Figure S11. Positive mode ESI-QTOF mass spectra of the self-cleaved $C_{cleaved}$-domain from mutation-2. During and after self-cleaving the sample solution represents a mixture of pro- and active *Md*PPO1 as well as the free C-terminal domain. However, the C-terminal domain was ionized best and led to the here presented high-resolution spectra. The inset shows that two different species (A-B) were found and the second inset of the charge state [16+] shows that isotopic resolution was achieved. The two different species indicate self-cleavage at two different proteolytic sites within the sequence (His361-Ala362-Ala363). Species A corresponds to the fragment obtained upon cleavage between Ala362-Ala363, species B to cleavage between His361-Ala362.

Species	M calculated Da	M (measured) Da	Δ/Da
A	15550.27	15550.59	-0.32
B	15621.83	15621.67	+0.16

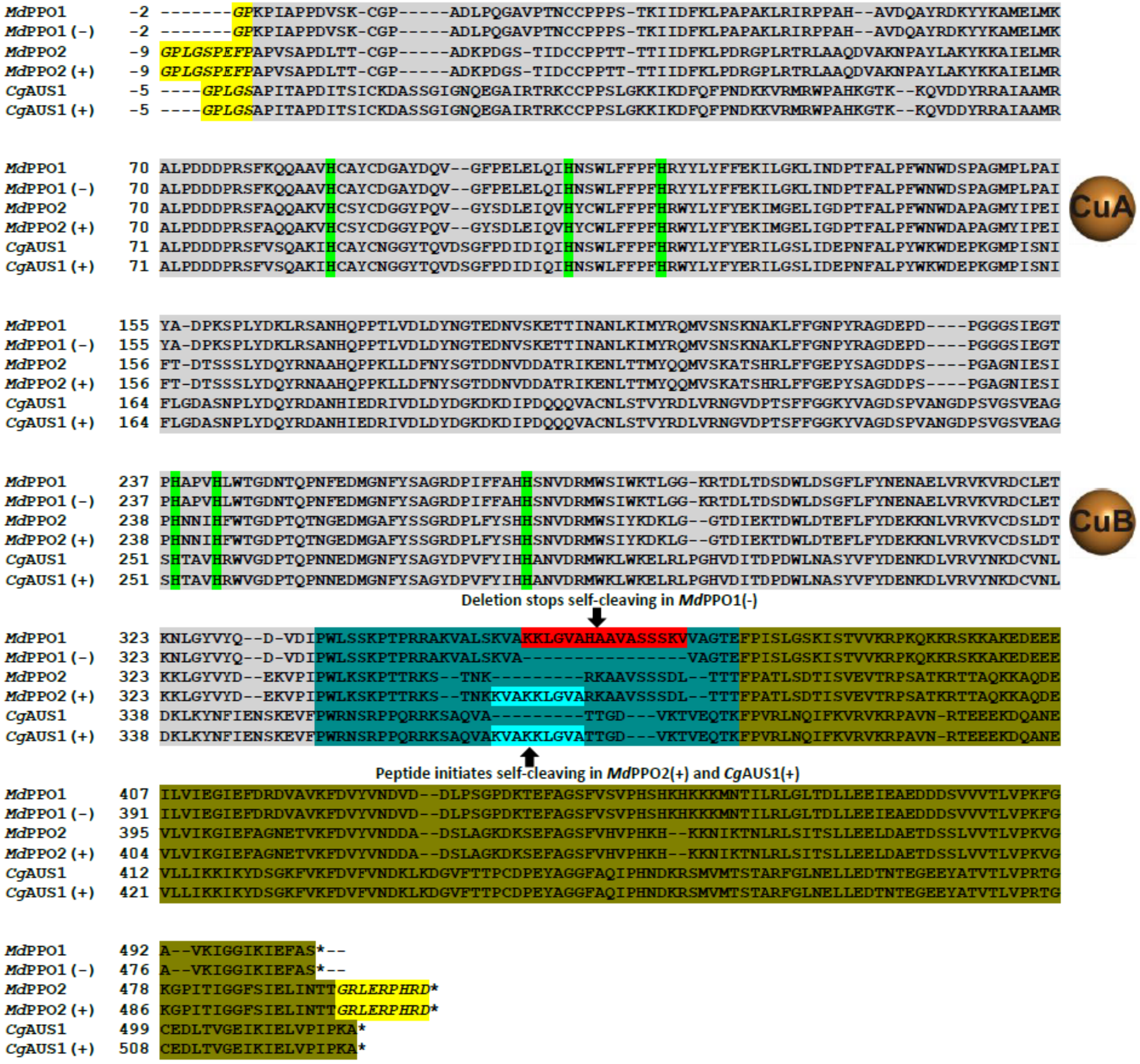

Figure S12. Sequence alignment of *Md*PPO1, *Md*PPO2, *Cg*AUS1 and the mutants *Md*PPO1(-), *Md*PPO2(+) and *Cg*AUS1(+). Highlighted are the conserved copper-coordinating histidines (green) of the dicopper centre. The deletion of the *Md*PPO1 (KKLGVAHAAVASSSKV) leads to the *Md*PPO1(-) which stops the self-cleavage and remains intact and from the other hand the insertion (KVAKKLGVA) to the *Md*PPO2 and *Cg*AUS1 lead to the *Md*PPO2(+) and *Cg*AUS1(+) mutants respectively which both exhibit self-cleavage reaction. The grey part of the sequence represents the active domain, the blue-green part represents the linker domain, while the olive part represents the C-terminal domain. The yellow parts at the start and the end of the sequences are remaining residues from the vector.

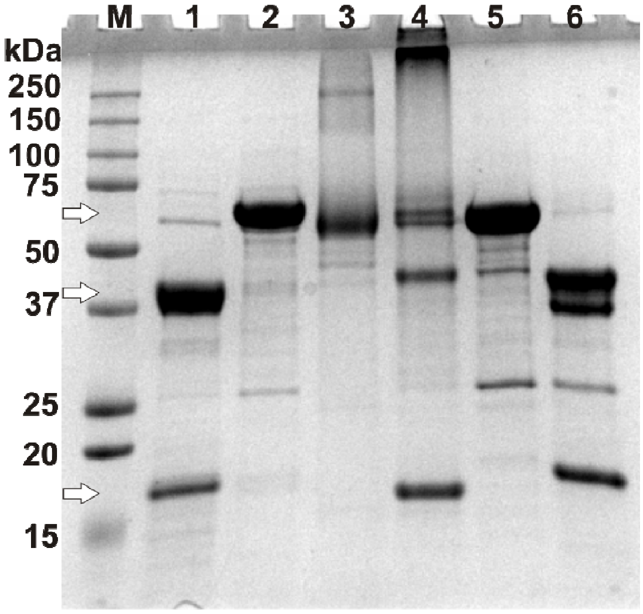

Figure S13. SDS-PAGE of *Md*PPO1, *Md*PPO2 and *Cg*AUS1 and the respective mutants *Md*PPO1(-), *Md*PPO2(+) and *Cg*AUS1(+). Lane 1) 10 µg of *Md*PPO1 upon self-cleavage resulting in the appearance of the active and the C-terminal domain ($C_{cleaved}$-domain), lane 2) 10 µg of *Md*PPO1(-) mutant which remains intact, lane 3) 10 µg of *Md*PPO2 does not exhibit any cleavage event, lane 4) 10 µg of *Md*PPO2(+) mutants which initiates the self-cleaving, lane 5) 10 µg of *Cg*AUS1 does not exhibit any cleavage and lane 6) 10 µg of *Cg*AUS1(+) mutant which initiates the self-cleaving event. All the samples were applied to SDS-PAGE under reduced conditions. M indicates the marker (molecular masses are given in kDa).

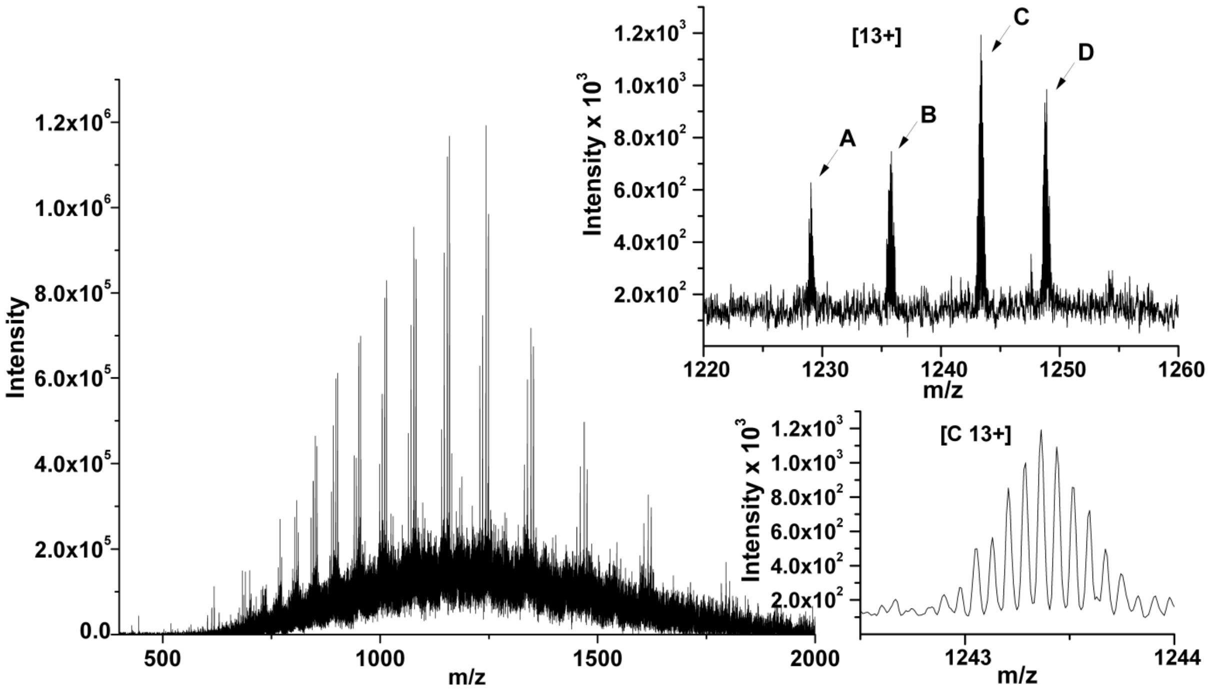

Figure S14. Positive mode ESI-LTQ-Orbitrap Velos of the self-cleaved $C_{cleaved}$-domain from *Md*PPO2(+) mutation. During and after self-cleavage the sample solution represents a mixture of pro- and active *Md*PPO2 as well as the free C-terminal domain. The C-terminal domain was ionized best and led to the here presented high-resolution spectra. The inset shows that two different species (A-B-C-D) were found and the second inset of the charge state [13+] shows that isotopic resolution was achieved. The four different species indicate self-cleavage at two different sites within the sequence (Ala362-Ala363-Val364-Ser365-S366). Species A corresponds to the fragment obtained upon cleavage between -Ser366, species B to cleavage between Val364-Ser365, species C to the cleavage between Ala363-Val364 and species D to the cleavage between Ala362-Ala363.

Species	M calculated Da	M (measured) Da	Δ/Da
A	15964.81	15964.54	-0.27
B	16051.89	16051.54	-0.35
C	16151.02	16151.61	+0.59
D	16222.10	16221.65	-0.45

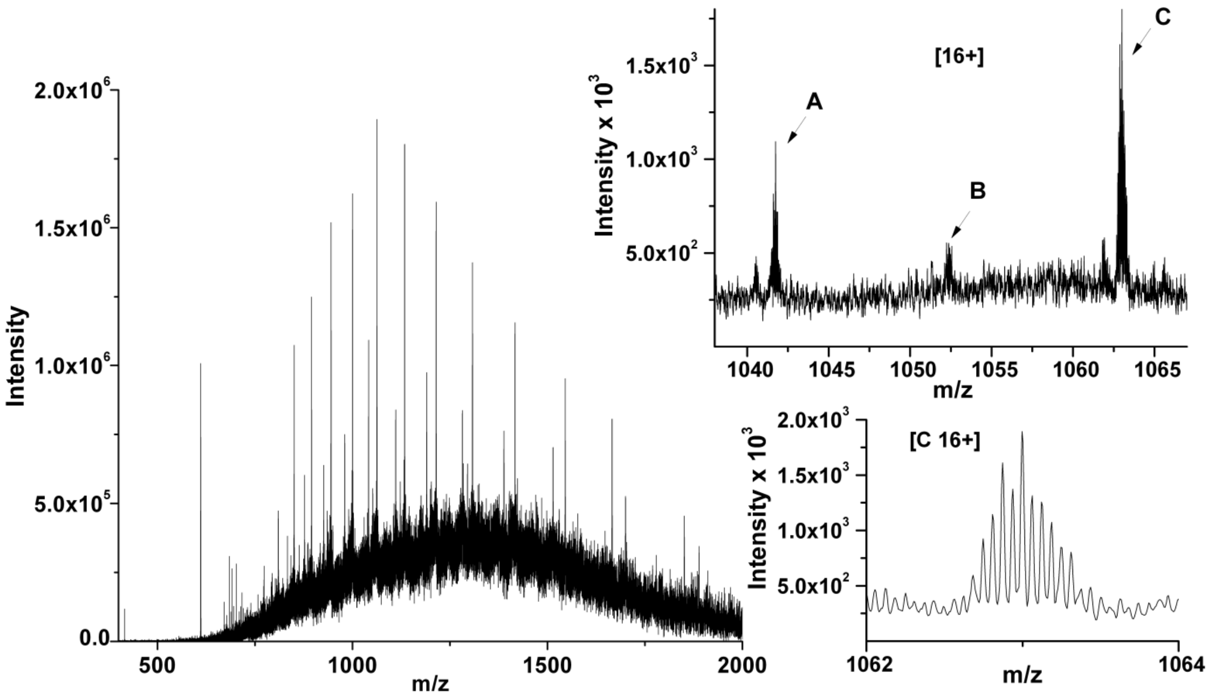

Figure S15. Positive mode ESI-LTQ-Orbitrap Velos of the self-cleaved $C_{cleaved}$-domain from *Cg*AUS1(+) mutant. During and after self-cleaving the sample solution represents a mixture of pro- and active *Cg*AUS1(+) as well as the C-terminal domain. The C-terminal domain was ionized best and led to the here presented high-resolution spectra. The inset shows that two different species (A-B-C) were found and the second inset of the charge state [16+] shows that isotopic resolution was achieved. The three different species indicate self-cleavage at different sites within the sequence (Lys374-Leu375-Gly376-Val377-Ala378-Thr379). Species A corresponds to the fragment obtained upon cleavage between Ala378- species B to cleavage between Gly376-Val377, species C to the cleavage between Lys374-Leu375.

Species	M calculated Da	M (measured) Da	Δ/Da
A	16651.84	16651.67	-0.17
B	16822.04	16821.69	-0.41
C	16992.25	16991.84	-0.36

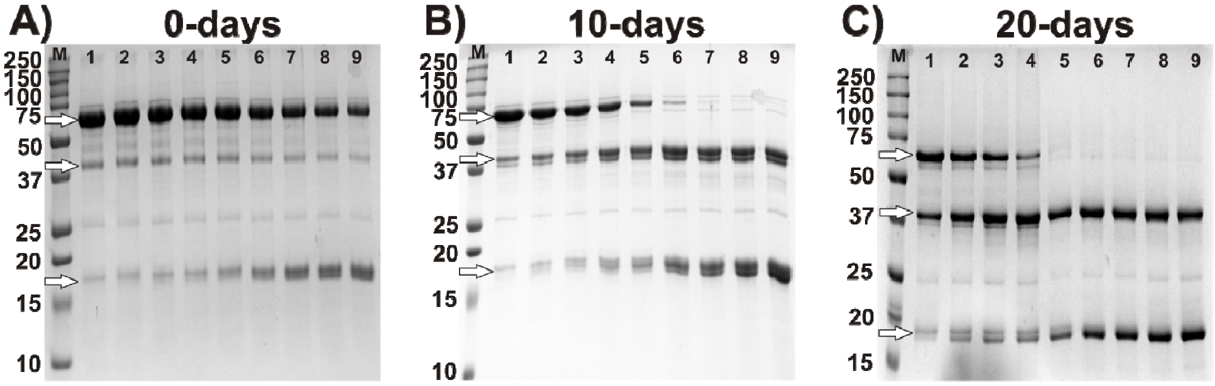

Figure S16. SDS-PAGE of *Md*PPO1 incubated with external C-terminal domain (C_{sole}-domain) for different time intervals. In every lane 7,5 µg of the latent *Md*PPO1 were applied. A-C show gels obtained after different incubation times as indicated above the respective gels Lane 1) 300 µg of *Md*PPO1 with 0 µg C_{sole}-domain, lane 2) 300 µg of *Md*PPO1 with 10 µg C_{sole}-domain, lane 3) 300 µg of *Md*PPO1 with 20 µg C_{sole}-domain, lane 4) 300 µg of *Md*PPO1 with 40 µg C_{sole}-domain, lane 5) 300 µg of *Md*PPO1 with 80 µg C_{sole}-domain, lane 6) 300 µg of *Md*PPO1 with 200 µg C_{sole}-domain, lane 7) 300 µg of *Md*PPO1 with 400 µg C_{sole}-domain, lane 8) 300 µg of *Md*PPO1 with 600 µg C_{sole}-domain and lane 9) 300 µg of *Md*PPO1 with 900 µg C_{sole}-domain. M indicates the molecular marker (molecular masses in kDa). Pro-*Md*PPO1 and C_{sole}-domain were mixed and incubated at 4°C. After only one day the mixtures containing higher amounts of C_{sole}-domain (lanes 7, 8 and 9) exhibit remarkably higher proteolytic activity than those with lower amounts of C_{sole}-domain. After 4 to 7 days, *Md*PPO1 is almost completely cleaved in the samples containing high amounts of C_{sole}-domain (lanes 7, 8 and 9). The arrows indicate the positions of the pro-*Md*PPO1 (top), the active enzyme (middle) and the C-terminal domain (bottom).

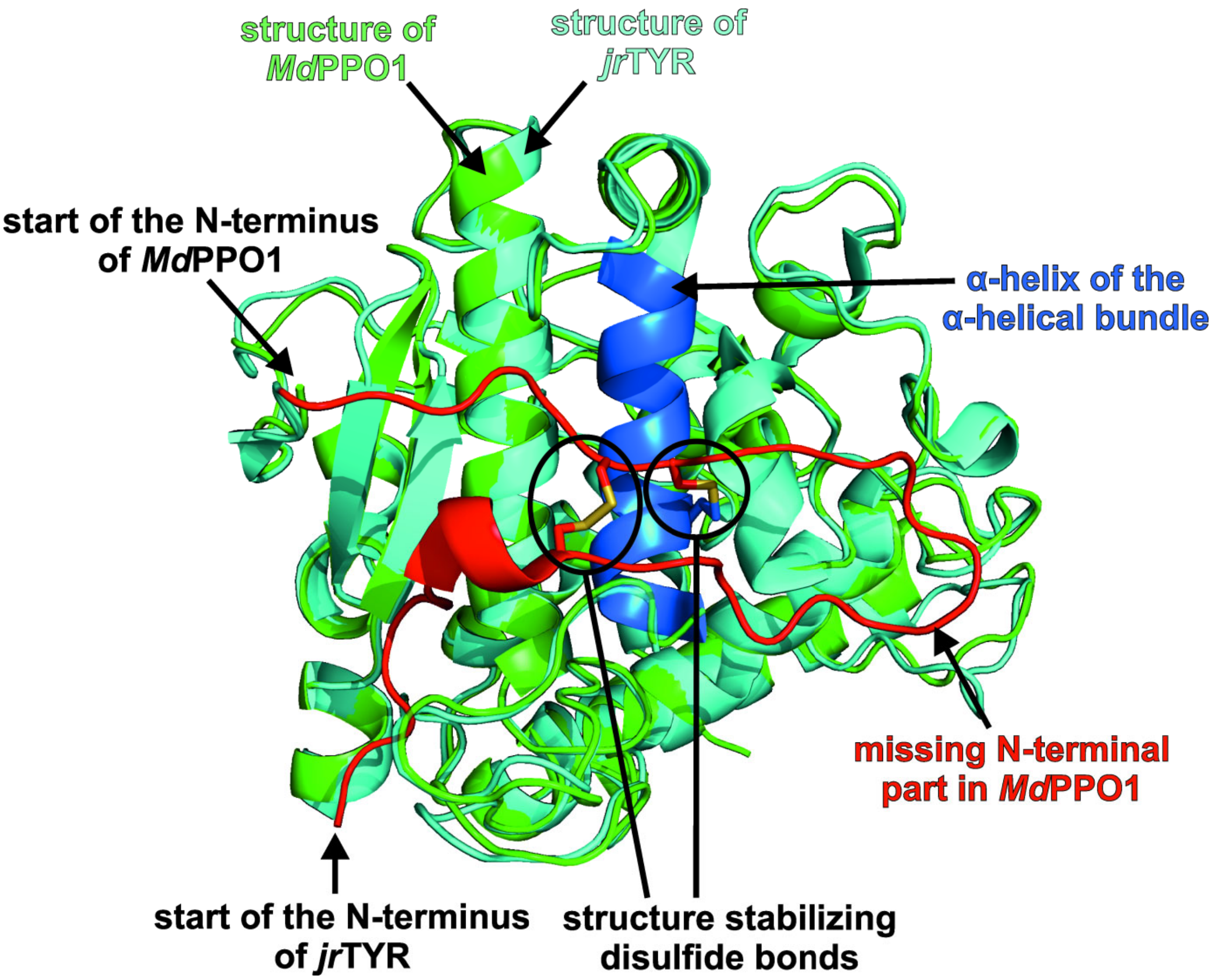

Figure S17. Structural alignment of *Md*PPO1 (PDB 6ELS)[5] and *jr*TYR (PDB 5CE9)[10,11]. The main domains of the two tyrosinases have been aligned. The N-terminal part of *jr*TYR (31 amino acids), which is missing in *Md*PPO1, is highlighted in red color. The two conserved disulfide bonds are marked by black color (circles). The N-terminal part in the structure of the pro-*Md*PPO1 was not obvious due to the lack of electron density. Our assumption for why the N-terminal part is missing is the lack of the conserved disulfide bonds in *Md*PPO1 (Cys11-Cys26 and Cys25-Cys87). In other plant PPOs like *jr*TYR, the N-terminal part embraces one α-helix of the tetrahelical bundle, which is highlighted in blue. The lack of the stabilizing disulfide bonds leads to a flexible N-terminal part and hence to a compromised order of the respective region.

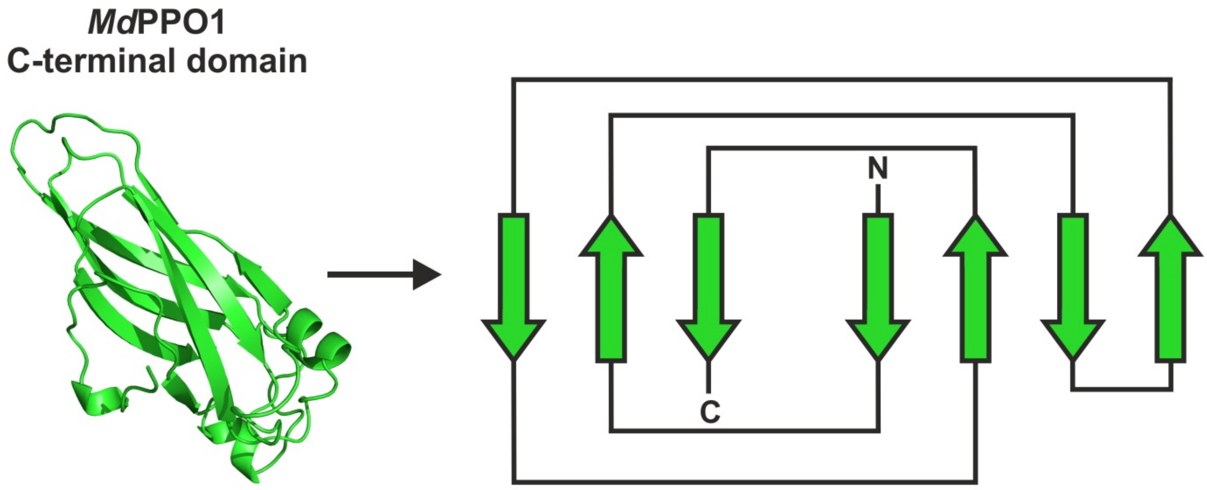

Figure S18. Structural topology of the $C_{cleaved}$-domain (PDB 6ELT) and the C_{sole}-domain (PDB 6ELV).

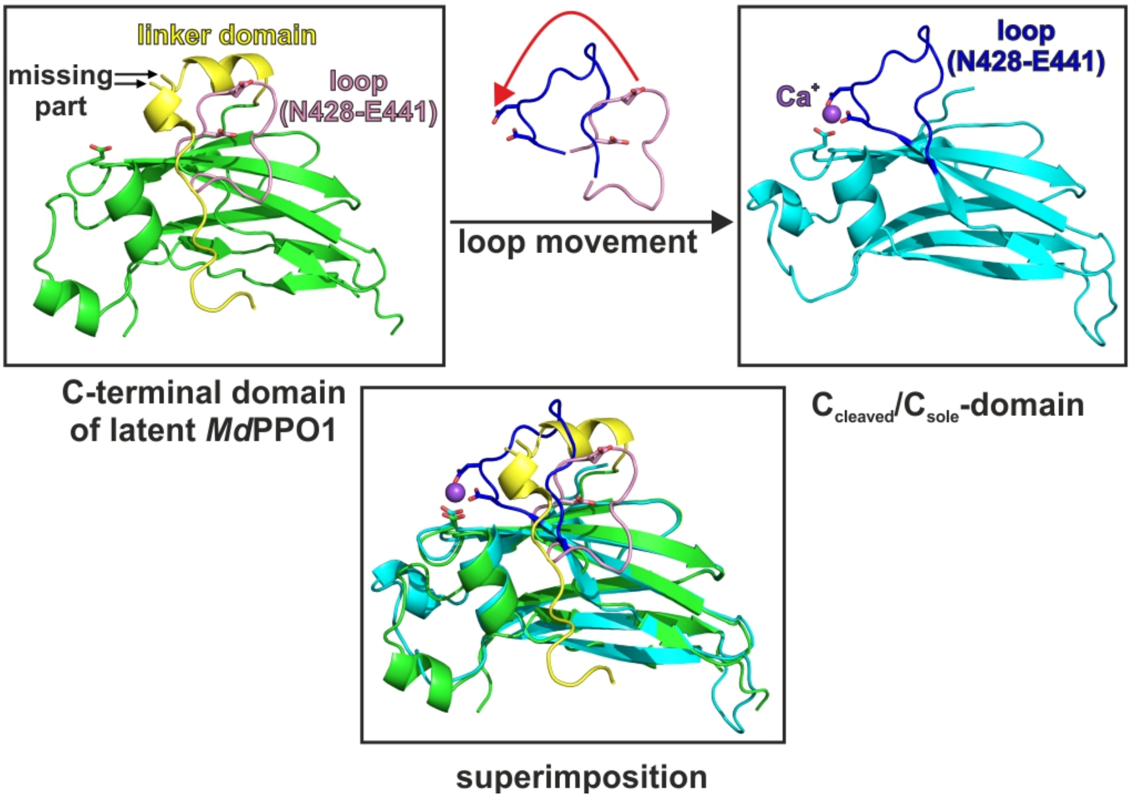

Figure S19. Comparison between the C-terminal domain of pro-*Md*PPO1 and the separated C-terminal domain $C_{cleaved}$ / C_{sole} The figure illustrates the movement of the loop (Asn428-Thr440) by indicating its position in the C-terminal structure of the pro-enzyme (left, domain in green and loop in pink) and in the separated C-terminal structure (right, domain in cyan and loop in dark blue). This loop movement leads to the formation of the Ca^{2+} binding site in the $C_{cleaved}$- and C_{sole}-domain, respectively. A superimposition of both C-terminal structures is shown in the middle (pink loop = loop position in the C-terminal domain of the pro-enzyme; blue loop = loop position in the separated C-terminal domain).

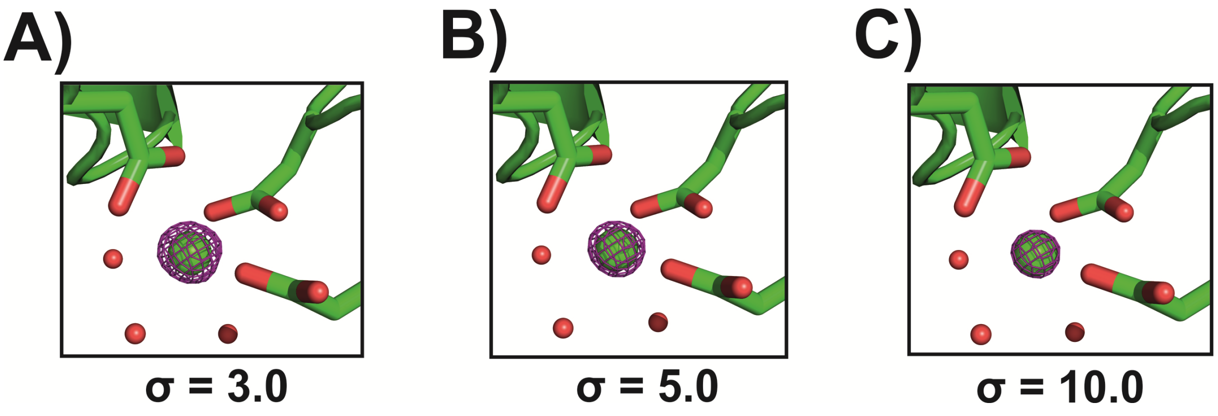

Figure S20. Identification of the bound metals in the $C_{cleaved}$- and C_{sole}-domain. **A) - C)** show the crystal structure of the C_{sole}-domain as green cartoon, whereas the anomalous signal of the bound metal (cyan sphere) is shown by an anomalous difference map (purple mesh), contoured at 3.0, 5.0 and 10.0 sigma, respectively. Based on these results and the composition of the used media and buffers calcium was identified as the metal binding site in both structures.

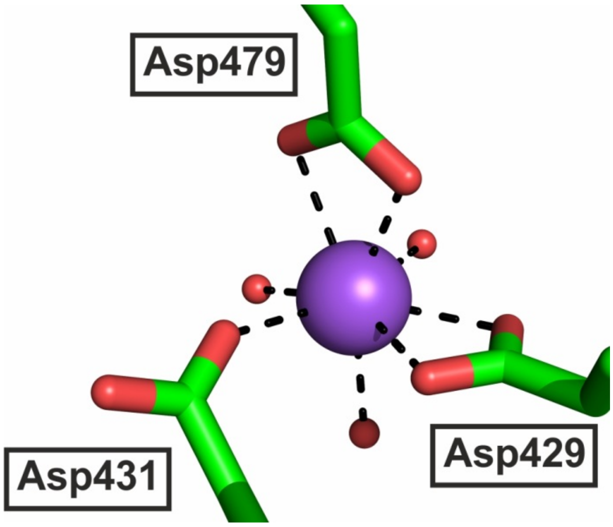

Figure S21. Geometry of the Ca^{2+} binding in the $C_{cleaved}$- and C_{sole}-domain. The Ca^{2+} ion coordinates to eight O-donor ligands, three aspartate residues (Asp429, Asp431 and Asp479) and three water molecules, whereby two of these aspartates (Asp429 and Asp479) act as bidentate ligands.

4. Supplementary Tables

Table S1. Mutations in *Md*PPO1, *Md*PPO2 and *Cg*AUS1 and the corresponding cleavage sites.

	Enzyme	Target	Mutation	Result	Cleavage site
1	*Md*PPO1	-	-	Self-cleavage	S'S'S'K'V $_{(365\text{-}369)}$
2	*Md*PPO1-mut1	SSKV $_{(366\text{-}369)}$	IDGR	Self-cleavage	K'K'L $_{(355\text{-}357)}$
3	*Md*PPO1	KKL $_{(355\text{-}357)}$	GAG	Self-cleavage	H'A'AVA'S'S $_{(360\text{-}366)}$
4	*Md*PPO1-mut2	KKL / SSKV $_{(355\text{-}357)\ /\ (366\text{-}369)}$	GAG / IDGR	Self-cleavage	H'A'A $_{(359\text{-}362)}$
5	*Md*PPO1	KVAKKLGVAH $_{(352\text{-}361)}$	deletion	Self-cleavage	S'S $_{(356\text{-}357)}$
6	*Md*PPO1	KVAKKLGVAH / S_{435} $_{(352\text{-}361)\ /\ 435}$	deletion / G_{435}	Self-cleavage	S'S $_{(356\text{-}357)}$
7	*Md*PPO1	DVDDLPSGP $_{(429\text{-}437)}$	deletion	Self-cleavage	A'S'SSK'V $_{(365\text{-}370)}$
8	*Md*PPO1	DVDDLPSGP $_{(429\text{-}437)}$ / KVAKKLGVAH $_{(352\text{-}361)}$	deletion	Self-cleavage	S'AAVAS'SSK'V $_{(351\text{-}360)}$
9	*Md*PPO1	S_{385}	G_{385}	Self-cleavage	A'S'S $_{(365\text{-}367)}$
10	*Md*PPO1	D_{429} / D_{431}	G_{429} / G_{431}	Self-cleavage	S'K'V $_{(367\text{-}369)}$
11	*Md*PPO1	S_{435}	G_{435}	Self-cleavage	S'SS'K'V $_{(365\text{-}369)}$
12	*Md*PPO1	D_{429} / D_{431} / S_{435}	G_{429} / G_{431} / G_{435}	Self-cleavage	K'L $_{(356\text{-}357)}$
13	*Md*PPO1	D_{479}	G_{479}	Self-cleavage	K'L $_{(356\text{-}357)}$
14	MdPPO1(-)	KKLGVAHAAVASSSKVV $_{(355\text{-}371)}$	deletion	Intact	-
15	*Md*PPO2	-	-	Intact	-
16	*Md*PPO2(+)	K_{350}-R_{351} - Insertion in-between	KVAKKLGVA	Self-cleavage	A'A'V'S'S $_{(362\text{-}366)}$
17	*Cg*AUS1	-	-	Intact	-
18	*Cg*AUS1(+)	A_{369}-T_{370} - Insertion in-between	KVAKKLGVA	Self-cleavage	K'LG'VA'T $_{(374\text{-}379)}$

Table S2. Primers for the construction of the C_{sole}-domain of *Md*PPO1 in pGEX-6P-1 and the mutants of *Md*PPO1, *Md*PPO2 and *Cg*AUS1.

Mutation	Primers	Template
C_{sole}-domain	fw 5´ GCAGCTGTTGCGTCGTC 3´<br>rev 5´ GGGCCCCTGGAACAGAAC 3´	*Md*PPO1 (pGEX-6P-1)
Mutation-1<br>SSKV $_{(366\text{-}369)}$ - IDGR	fw 5´ gggagaGTGGCAGGCACTGAGTTC 3´<br>rev 5´ gtcgatCGACGCAACAGCTGCGTG 3´	*Md*PPO1 (pGEX-6P-1)
Mutation-2<br>KKL $_{(355\text{-}357)}$ - GAG	fw 5´ tggtGGAGTTGCACACGCAGCT 3´<br>rev 5´ gcaccCGCTACTTTGCTCAATGCAAC 3´	*Md*PPO1 (pGEX-6P-1)
Mutation-3<br>KKL / SSKV $_{(355\text{-}357)}$ / $_{(366\text{-}369)}$ - GAG / IDGR	fw 5´ tggtGGAGTTGCACACGCAGCT 3´<br>rev 5´ gcaccCGCTACTTTGCTCAATGCAAC 3´	*Md*PPO1 mutation-1
Mutation-4<br>KVAKKLGVAH $_{(352\text{-}361)}$ / deletion	fw 5´ GCAGCTGTTGCGTCGTCC 3´<br>rev 5´ GCTCAATGCAACTTTCGCCC 3´	*Md*PPO1 (pGEX-6P-1)
Mutation-5<br>KVAKKLGVAH $_{(352\text{-}361)}$ / S_{435} - deletion / G_{435}	fw 5´ TGACTTGCCGggtGGGC 3´<br>rev 5´ TCGACGTCATTCACATACACATCAAACTTCA 3´	*Md*PPO1 mutation-4
Mutation-6<br>DVDDLPSGP $_{(429\text{-}437)}$ - deletion	fw 5´ GACAAGACGGAGTTTGCC 3´<br>rev 5´ ATTCACATACACATCAAACTTCAC 3´	*Md*PPO1 (pGEX-6P-1)
Mutation-7<br>DVDDLPSGP $_{(429\text{-}437)}$ / KVAKKLGVAH $_{(352\text{-}361)}$ - deletions	fw 5´ GACAAGACGGAGTTTGCC 3´<br>rev 5´ ATTCACATACACATCAAACTTCAC 3´	*Md*PPO1 mutation-4
Mutation-8<br>S_{385} - G_{385}	fw 5´ GTCGAAGATAgggACGGTGGTGA 3´<br>rev 5´ CCCAGACTTATCGGGAAC 3´	*Md*PPO1 (pGEX-6P-1)
Mutation-9<br>D_{429} / D_{431} – G_{429} / G_{431}	fw 5´ GTATGTGAATGgCGTCGgTGACT 3´<br>rev 5´ ACATCAAACTTCACAGCCACG 3´	*Md*PPO1 (pGEX-6P-1)
Mutation-10<br>S_{435} – G_{435}	fw 5´ TGACTTGCCGggtGGGC 3´<br>rev 5´ TCGACGTCATTCACATACACATCAAACTTCA 3´	*Md*PPO1 (pGEX-6P-1)
Mutation-11<br>D_{429} / D_{431} / S_{435} – G_{429} / G_{431} / G_{435}	fw 5´ TGACTTGCCGggtGGGCCT 3´<br>rev 5´ ccgACGccaTTCACATACACATCAAAC 3´	*Md*PPO1 (pGEX-6P-1)
Mutation-12<br>D_{479} / G_{479}	fw 5´ GGCGGAGGACggtGACA 3´<br>rev 5´ TCAATTTCCTCCAACAAATCTGTCAAC 3´	*Md*PPO1 (pGEX-6P-1)
Mutation-13<br>KKLGVAHAAVASSSKVV $_{(355\text{-}371)}$ - deletion	fw 5´ GTGGCAGGCACTGAGTTC 3´<br>rev 5´ CGCTACTTTGCTCAATGC 3´	*Md*PPO1 (pGEX-6P-1)
Mutation-14<br>K_{350}-R_{351} - Insertion KVAKKLGVA	fw 5´ gctgggagttgcaAGAAAGGCCGCAGTTTCA 3´<br>rev 5´ ttcttcgctactttCTTATTCGTCGACTTACGAG 3´	*Md*PPO2 (pGEX-6P-1)
Mutation-15<br>A_{369}-T_{370} - Insertion KVAKKLGVA	fw 5´ gctgggagttgcaACGACTGGAGATGTGAAGAC 3´<br>rev 5´ ttcttcgctactttCGCAACCTGGGCACTCTT 3´	*Cg*AUS1 (pGEX-6P-1)

Table S3. Data collection and processing of pro-*Md*PPO1, $C_{cleaved}$-domain and C_{sole}-domain.

	Pro-*Md*PPO1 (PDB 6ELS)	$C_{cleaved}$-domain (PDB 6ELT)	C_{sole}-domain (PDB 6ELV)
**Crystal data and data collection**			
Diffraction source	ID-23, ESRF	ID-30A-1, ESRF	ID-23-1, ESRF
Wavelength (Å)	0.9724	0.9660	0.9724
Temperature (K)	100	100	100
Detector	PILATUS 6M	PILATUS 2M	PILATUS 6M
Crystal-detector distance (mm)	190	116	280
Rotation range per image (°)	0.1	0.15	0.15
Total rotation range (°)	320	125.1	360
Exposure time per image (s)	0.125	0.06	0.045
Space group	$P2_12_12_1$	$P2_12_12_1$	$P2_12_12_1$
a, *b*, *c* (Å)	50.70, 80.15, 115.96	45.23, 50.14, 50.72	45.13, 50.10, 50.54
Mosaicity (°)	0.173	0.15	0.08
Resolution range (Å)	46.45 - 1.346	35.66 - 1.35	35.58 - 1.05
Total No. of reflections	1214526 (117878)	117282 (11826)	577356 (16715)
No. of unique reflections	104709 (10123)	25835 (1281)	51007 (3321)
Completeness (%)	99.51 (97.62)	99.0 (100.00)	94 (62.00)
Redundancy	11.6 (11.6)	4.5 (4.7)	11.3 (50)
$\langle I/\sigma(I)\rangle$	11.72 (2.02)	10.41 (1.74)	20.85 (2.72)
$R_{r.i.m.}$	0.12 (1.174)	0.093 (0.937)	0.016 (0.248)
$R_{p.i.m}$†	0.034 (0.337)	0.042 (0.422)	0.058 (0.572)
$CC_{1/2}$#	0.996 (0.708)	0.998 (0.659)	0.999 (0.837)
Overall *B* factor from Wilson plot (Å^2)	14.00	12.07	10.30
**Refinement statistics**			
Resolution range (Å)	46.45 - 1.35	35.66 - 1.35	35.58 - 1.05
No. of unique reflections refined against	104707 (10123)	25834	51002
R_{work}* (%)	14.29 (28.52)	17.09	14.76
R_{free}** (%)	17.93	20.10	16.07
Average B-factor (Å^2)	19.00	17.00	16.00

† $R_{p.i.m.} = \Sigma_{hkl}\{1/[(N(hkl)-1]\}^{1/2}\ \Sigma_i|I_i(hkl)-\langle I(hkl)\rangle|/\Sigma_{hkl}\Sigma_i I_i(hkl)$, where $I_i(hkl)$ is the ith observation of reflection hkl and $\langle I(hkl)\rangle$ is the weighted average intensity for all observations of reflection hkl.

# $CC_{1/2}$ is defined as the correlation coefficient between two random half data sets.

* $R_{work}=\Sigma|F_{calcd}|-|F_{obs}|/\Sigma|F_{obs}s|$, where F_{calcd} and F_{obs} are the calculated and observed structure factor amplitudes, respectively

** R_{free} is calculated for a randomly chosen 5 % of the reflections within each dataset.

5. References

[1] I. Kampatsikas, A. Bijelic, M. Pretzler, A. Rompel, *Sci. Rep.* **2017**, *7*, 8860.

[2] U. K. Laemmli, *Nature* **1970**, *227*, 680–685.

[3] D. F. Swinehart, *J. Chem. Educ.* **1962**, *39*, 333.

[4] E. Gasteiger, C. Hoogland, A. Gattiker, S. Duvaud, M. Wilkins, R. Appel, A. Bairoch, in *Proteomics Protoc. Handb.* (Ed.: J. Walker), Humana Press, **2005**, pp. 571–607.

[5] I. Kampatsikas, A. Bijelic, M. Pretzler, A. Rompel, *Acta Crystallogr. Sect. F Struct. Biol. Commun.* **2017**, *73*, 491–499.

[6] W. Kabsch, *Acta Crystallogr. D Biol. Crystallogr.* **2010**, *66*, 125–132.

[7] S. F. Altschul, W. Gish, W. Miller, E. W. Myers, D. J. Lipman, *J. Mol. Biol.* **1990**, *215*, 403–410.

[8] C. Molitor, S. G. Mauracher, A. Rompel, *Acta Crystallogr. Sect. F Struct. Biol. Commun.* **2015**, *71*, 746–751.

[9] C. Molitor, S. G. Mauracher, A. Rompel, *Proc. Natl. Acad. Sci. U. S. A.* **2016**, *113*, E1806–E1815.

[10] F. Zekiri, A. Bijelic, C. Molitor, A. Rompel, *Acta Crystallogr. Sect. F Struct. Biol. Commun.* **2014**, *70*, 832–834.

[11] A. Bijelic, M. Pretzler, C. Molitor, F. Zekiri, A. Rompel, *Angew. Chem. Int. Ed.* **2015**, *54*, 14677–14680.

[12] P. D. Adams, P. V. Afonine, G. Bunkóczi, V. B. Chen, I. W. Davis, N. Echols, J. J. Headd, L.-W. Hung, G. J. Kapral, R. W. Grosse-Kunstleve, et al., *Acta Crystallogr. D Biol. Crystallogr.* **2010**, *66*, 213–221.

[13] A. Šali, T. L. Blundell, *J. Mol. Biol.* **1993**, *234*, 779–815.

[14] T. C. Terwilliger, R. W. Grosse-Kunstleve, P. V. Afonine, N. W. Moriarty, P. H. Zwart, L.-W. Hung, R. J. Read, P. D. Adams, *Acta Crystallogr. D Biol. Crystallogr.* **2008**, *64*, 61–69.

[15] E. Krissinel, K. Henrick, *J. Mol. Biol.* **2007**, *372*, 774–797.

[16] M. Pretzler, A. Bijelic, A. Rompel, in *Ref. Module Chem. Mol. Sci. Chem. Eng.*, Elsevier, **2015**. doi: 10.1016/B978-0-12-409547-2.11521-5

[17] M. Pretzler, A. Bijelic, A. Rompel, *Sci. Rep.* **2017**, *7*, 1810.

[18] S. G. Mauracher, C. Molitor, C. Michael, M. Kragl, A. Rizzi, A. Rompel, *Phytochemistry* **2014**, *99*, 14–25.

[19] E. Seiradake, A. Schaupp, D. del T. Ruiz, R. Kaufmann, N. Mitakidis, K. Harlos, A. R. Aricescu, R. Klein, E. Y. Jones, *Nat. Struct. Mol. Biol.* **2013**, *20*, 958–964.

[20] Ö. D. Ekici, M. Paetzel, R. E. Dalbey, *Protein Sci. Publ. Protein Soc.* **2008**, *17*, 2023–2037.

[21] M. Paetzel, *Biochim. Biophys. Acta BBA - Mol. Cell Res.* **2014**, *1843*, 1497–1508.
